# Sex-differences in associations of LV structure and function measured by echocardiography with long-term risk of mortality and cardiovascular morbidity

**DOI:** 10.3389/fcvm.2023.1144964

**Published:** 2023-04-25

**Authors:** Lamia Al Saikhan, Chloe Park, Therese Tillin, Siana Jones, Darrel Francis, Jamil Mayet, Nish Chaturvedi, Alun D. Hughes

**Affiliations:** ^1^ Department of Cardiac Technology, College of Applied Medial Sciences, Imam Abdulrahman Bin Faisal University, Dammam, Saudi Arabia; ^2^ MRC Unit for Lifelong Health and Ageing, UCL Institute of Cardiovascular Science, University College London, London, United Kingdom; ^3^ National Heart and Lung Institute, Imperial College London, London, United Kingdom

**Keywords:** 3D echocardiography, sex-differences, all-cause mortality, LV remodeling, cardiovascular morbidity

## Abstract

**Background:**

Three-dimensional echocardiography (3DE) measures of the left ventricle (LV) predict outcomes in high risk individuals, but their prognostic value in the general population is unknown. We aimed to establish whether 3DE was associated with mortality and morbidity in a multi-ethnic community-based sample, if associations differed by sex, and explored potential mechanisms explaining sex differences.

**Methods:**

922 individuals (69.7 ± 6.2 years; 717 men) from the SABRE study underwent a health examination including echocardiography. Associations between 3DE LV measures (ejection fraction (EF), end-diastolic volume (EDV), end-systolic volume (ESV), LV remodeling index (LVRI) and LV sphericity index (LVSI), and all-cause mortality and a composite cardiovascular endpoint [comprising new onset (non)fatal coronary heart disease, heart failure hospitalization, new-onset arrhythmias and cardiovascular mortality] were determined using multivariable Cox regression over a median follow-up of 8 years (all-cause mortality) and 7 years (composite cardiovascular endpoint).

**Results:**

There were 123 deaths and 151 composite cardiovascular endpoints. Lower EF, higher LV volumes and LVSI were associated with increased all-cause mortality, and higher LV volumes were associated with the composite cardiovascular endpoint independent of potential confounders. Associations between LV volumes, LVRI, LVSI, and mortality differed by sex (*p* interaction <0.1). In men increased LV volumes and LVSI and decreased LVRI and EF were associated with higher mortality, but associations were null or reversed in women (hazard ratios (95% CI) men vs. women: EDV 1.25 (1.05, 1.48) vs. 0.54 (0.26, 1.10); ESV, 1.36 (1.12, 1.63) vs. 0.59 (0.33, 1.04); LVRI, 0.79 (0.64, 0.96) vs. 1.70 (1.03, 2.80); LVSI, 1.27 (1.05, 1.54) vs. 0.61 (0.32, 1.15); and EF, 0.78 (0.66, 0.93) vs. 1.27 (0.69, 2.33). Similar sex differences were observed for associations with the composite cardiovascular outcome. Adjustment for LV diastolic stiffness and arterial stiffness marginally attenuated these differences.

**Conclusions:**

3DE measures of LV volume and remodeling are associated with all-cause mortality and cardiovascular morbidity; however, some associations differ by sex. Sex-differences in LV remodeling patterns may influence mortality and morbidity risk in the general population.

## Introduction

Asymptomatic left ventricular (LV) systolic dysfunction is common among older community-dwelling individuals and predicts subsequent symptomatic heart failure (HF) and death (1). LV ejection fraction (EF) and volumes by 2D echocardiography (2DE) are widely used as prognostic indicators, although they suffer from limitations, being dependent on operator experience, the quality of the acquisition and geometric assumptions (2). 3D echocardiography (3DE) may be superior to 2DE as it makes no geometrical assumptions and is unaffected by foreshortening (2). The availability of 3DE has also led to an increasing interest in LV shape analysis, which may offer additional information (3). 3DE LV measures predict mortality and adverse cardiovascular events in various patient groups at high cardiovascular risk (3–5), however the possibility that sex modifies associations has not been explored, despite considerable evidence that LV structure and function differ by sex (6), and that diastolic dysfunction and HF with preserved EF are commoner in women (7). It has been proposed that higher ventriculo-arterial stiffness (8), and increased microvascular disease (9) may contribute to these sex-differences.

Accordingly, we sought to 1) examine associations between LV 3DE measures and the risk of subsequent mortality and incident cardiovascular morbidity in a community dwelling multi-ethnic sample of older people, and 2) investigate whether associations differed by sex, and 3) to explore the potential roles of LV stiffness, macro- and micro-vascular diseases in any sex-differences.

## Methods

### Study population

SABRE (Southall and Brent revisited) study has been described previously (10, 11). Briefly, all traceable surviving participants from the baseline studies (1988–1991) were invited to attend the second-wave of follow-up at St Mary's Hospital, London. Of 3,433 traceable survivors, 1,438 (42%) participants (69.6 ± 6 years) attended clinic in 2008–2011 (Supplementary Figure S1). 3DE LV datasets were obtained in a total of 1,001 participants of which 924 datasets were suitable for volumetric analysis and 897 were suitable for mass analysis (11). All investigations were performed in accordance with the Helsinki Declaration, written informed consent was obtained, and the study was approved by St Mary's Hospital Research Ethics Committee (07/H0712/109). Further details regarding SABRE can be found at https://mrc.ukri.org/research/facilities-and-resources-for-researchers/cohort-directory/southall-and-brent-revisited-sabre/. Requests to access the dataset from qualified researchers trained in human subject confidentiality protocols may be sent to the MRC Unit for Lifelong Health and Ageing at UCL (sabre@ucl.ac.uk).

### Clinical examination

Participants underwent comprehensive investigations including anthropometrics, ECG, echocardiography, vascular, hemodynamic, biomedical measurements, and a health/lifestyle questionnaire (10, 11). Hypertension and diabetes mellitus (DM) were defined based on self-report, health records review or use of relevant medications. Fasting blood samples were drawn and analyzed for biomarkers including glucose, lipids (enzymatic methods), glycated hemoglobin-HbA1C (high-pressure liquid chromatography), C-reactive protein (automated analyzer), high-sensitive cardiac Troponin T and NT-ProBNP [Elecsys 2010 electrochemiluminescence analyzer (Roche Diagnostics, Burgess Hill, UK)]. eGFR was estimated using a validated equation based on serum Cystatin-C (12). An early morning urine sample was used to measure urinary albumin (enzyme immunoassay) and creatinine (Jaffe method), and albumin:creatinine ratio (ACR) was calculated. Microalbuminuria was defined according to the National Institute for Health and Care Excellence guidelines as follows: microalbuminuria (ACR 3–69 mg/mmol) and proteinuria (ACR ≥ 70 mg/mmol) (13). Retinopathy was defined according to the NHS Diabetic Eye Screening Programme (14), and was classified as follows: none, mild (non-proliferative disease), or moderate (proliferative) disease. There were no cases of severe proliferative disease, so this category was not used.

### Echocardiographic imaging and analysis

Transthoracic echocardiography was performed by two experienced sonographers using a Phillips iE33 equipped with S5-1 phased array and X3-1 matrix array transducers according to a standardized protocol (11).

### Conventional echocardiography

Chamber dimensions and wall thickness from 2D-guided M-mode were measured from parasternal long-axis view from which LV mass was calculated, following ASE recommendations (2). LV hypertrophy (LVH) was defined as LV mass indexed to body surface area (BSA) >115 g/m^2^ in men and 95 g/m^2^ in women (2). LV volumes were calculated by the Teichholz formula. This method is now considered sub-optimal, but when the study was designed it was consistent with contemporary guidelines, and its use maintained compatibility with methods used for previous visits (2). Tissue-Doppler analysis of lateral and septal mitral annulus motion was performed and peak longitudinal systolic velocity (s’), and peak early and late mitral annular relaxation velocities (e′, a′) were calculated and averaged. Mitral early and late diastolic inflow velocities and deceleration time were measured by PW Doppler with a sample volume placed at the tip of mitral valve leaflets from which the E/A ratio was calculated. E/e′ was calculated as an index of LV filling pressure and a marker of LV diastolic dysfunction (15).

### 3D echocardiography

LV full-volume 3D datasets of 4 sub-volumes (wide-angled 93° × 80°) were obtained from the apical position over 4 cardiac cycles. 3DE analysis was performed offline by two experienced sonographers using Philips QLAB software 7.0 with 3DQ for LV mass and 3DQ Advanced for LV volumes according to a pre-developed protocol and blinded to all other information (11). Cardiac 3DQ calculated LV mass using the biplane method of discs, and 3DQ Advanced measured 3D LV volumes without the geometrical assumptions of 2D methods (11). LV functional and structural measures were as follows: EF, end-diastolic (EDV), end-systolic (ESV), and stroke (SV) volumes indexed to BSA, LV mass indexed to BSA, and LV remodeling index (LVRI calculated as mass to EDV ratio) (16). LV sphericity index (LVSI) was calculated as EDV/spherical volume where spherical volume = 4/3∗π∗(D/2)^3^ (D = major end-diastolic LV long axis.) (17). 3DE-derived LV diastolic elastance (Ed) was calculated as (E/e′)/(EDV) (7). Indexation to height^2.7^ was also performed as a sensitivity analysis and LVH was defined as >45.1 g/m^2.7^ in men and 38.0 g/m^2.7^ in women (18). 3DE LVRI was used to determine LV geometry as follows: normal (LVRI ≤1.5 and no LVH); LV remodeling (LVRI >1.5 and no LVH); LV concentric hypertrophy (LVRI >1.5 and LVH); and LV eccentric hypertrophy (LVRI ≤1.5 and LVH) (7). Reproducibility of 3DE LV measures in SABRE population was previously reported (11).

### Vascular assessment

Three sitting blood pressure (BP) measurements were recorded in clinic using an Omron CEP 7,050 after resting for 15-minutes and the average of the last two readings was used. Central BP was assessed non-invasively using a SphygmoCor device (Atcor, Sydney, Australia) and calibrated to brachial BP according to manufacturer's instructions. Total arterial compliance (TAC) was calculated as SV/pulse pressure indexed to BSA and effective arterial elastance (Ea) was calculated as end-systolic pressure/SV indexed to BSA. Pulse wave velocity (PWV) was measured using a PT2000 device (Micro Medical Ltd, Kent).

### Follow-up and outcome assessment

Participants were followed for mortality and hospitalization from the time of the attended clinic (2008-2011) until December 2018 (mortality) and March 2017 (morbidity). Participants were flagged for death by the UK Office of National Statistics (ONS) and causes of deaths were defined according to the International Classification of Disease (ICD) diagnostic codes (ICD-10). Hospital Episode Statistics (HES), provided by NHS Digital, were used to provide information regarding admission, discharge and, the ICD diagnostic codes and operation codes [i.e., the Office of Population Censuses and Surveys (OPCS) classification of interventions and procedures].

Outcomes were all-cause mortality and a composite cardiovascular endpoint comprising new onset fatal and non-fatal coronary heart disease (CHD) (angina, myocardial infarction (MI) or its complications and atherosclerotic heart disease (ICD-9: 410–415, ICD-10: I200–I259), or emergent coronary revascularization interventions (OPCS codes: K401-K469, K491-K504, K751-K759)), HF hospitalization (ICD-9: 428, ICD-10: I50), new-onset atrial fibrillation (AF, ICD-9: 4,273, ICD-10: I48), ventricular fibrillation (ICD-9: 4,274, ICD-10: I490), and cardiovascular mortality (ICD-9: 390–460, 410–415 and 430–439, or ICD-10: I100-I990, I200-I259 and I600-I699). Cardiac dysfunction is an indicator of frailty in older people and multimorbidity can make the diagnosis of heart failure difficult, this may result in an underdiagnosis of heart failure in older people (19). This was the rational for choosing all-cause mortality as one of the outcomes of interest in this study. There were two deaths with an unknown year of death, and these were therefore excluded from the survival analysis. The final sample size included in this study was 922.

CHD events that occurred before SABRE clinic were identified from self-report and primary-care medical review. Medical record review was undertaken by trained research team members in local general practices and at primary care trusts. CHD events identified from primary care record review were adjudicated by two senior physicians according to the ASCOT study criteria (20).

### Statistical analysis

All analyses were performed using STATA 15.1 (StataCorp LLC, USA). Continuous variables are presented as mean ± SD or median (interquartile range). Categorical variables are presented as counts (percentages). Differences between two groups were assessed using a two-sample *t*-test with unequal variance or a *χ*^2^ test as appropriate. Survival analysis was performed using a Cox proportional hazard model, and hazard ratios (HR) with 95% confidence intervals (CI) are reported. Skewed variables were natural log transformed prior to inclusion in the Cox model. Cox regression model diagnostics were performed ensuring assumptions of proportional hazards were satisfied. Individuals were censored at the end of the follow-up, or at the time of event of interest. Non-cardiovascular death was considered a potential competing risk for composite cardiovascular endpoints and competing risk analysis using Fine-Gray estimators was performed as a sensitivity analysis.

Confounders were selected *a priori* based on Framingham cardiovascular risk factors (model-1: age, sex and ethnicity; model-2: model-1 plus systolic BP, antihypertensive medication, cholesterol: HDL ratio, body mass index (BMI), DM and smoking; additional models were run as sensitivity analyses adjusting for prior CHD. The possibility that prior CHD modified associations was investigated by testing for a statistical interaction and by excluding individuals with prior CHD. To assess whether sex modified associations, an interaction analysis by sex was performed, *p* < 0.1 was taken as suggestive of an interaction (21). All exposures were standardized to per standard deviation (SD) change to allow comparability between HR (ESV was log-transformed then standardized). Cumulative hazard curves were created stratified by sex using the Nelson-Aalen method and comparisons of events between strata were performed using the log-rank test. The median was used to dichotomize continuous predictive variables.

The role of markers of LV diastolic dysfunction (Ed), macro-vascular disease [TAC, Ea, augmentation index (AI) and PWV] and micro-vascular disease (eGFR, microalbuminuria and retinopathy) in explaining the possible sex-difference in associations between 3DE LV measures and outcomes was assessed. Each of these markers were added separately to a fully adjusted model (i.e., model-2; Supplementary Figure S2) for men and women separately. Sensitivity analysis was performed by replacing Ed with E/e′.

Missing exposure data were imputed using multiple imputation by chained equations (MICE), assuming that data were missing at random and twenty imputed datasets were created (22). The imputation models included all variables in fully adjusted analytical models as well as the event indicator and the estimate of the cumulative baseline hazard and additional variables (height, heart rate, EDV, antidiabetic medications and hypertension) that helped predict the missing covariates (22). Sensitivity analyses were performed using a complete case analysis for the associations between 3DE LV measures and outcomes for the overall population and stratified by sex.

A two-tailed *p*-value of <0.05 was considered statistically significant.

## Results

### Population characteristics

Characteristics of the study sample are summarized in Table 1. Mean age was 69.7 ± 6.2 years and 78% were male; men and women were similar in age. Men were taller and had larger waist to hip ratio, but women had a slightly higher BMI. Men were more likely to have DM and prior CHD. There were more male than female ex- and/or current smokers. Men had higher fasting total cholesterol: HDL ratio than women and were more likely to receive lipid-lowering therapy. Women had higher eGFR compared to men, but the prevalence of microalbuminuria and retinopathy were similar. Women had slightly higher NT ProBNP compared to men, but men had higher troponin. As expected, women had higher EF and smaller cavity volumes compared to men. Similarly, women had smaller LV mass than men, but not when indexed to height^2.7^. Both groups had similar LVRI and LVSI. Women had higher relative wall thickness and more concentric hypertrophy, but less concentric remodeling compared with men. While trans-mitral flow velocities were similar between the groups, women had evidence of poorer diastolic function (higher filling pressure and LV diastolic stiffness and lower e′ and a′ velocities than men). Women also had more poorer LV longitudinal systolic function (s′) than men. TAC was lower in women, while Ea was higher, but not after adjusting for BSA. AI was higher in women, but PWV was similar between the groups. Participants in whom 3DE analysis could not be performed were heavier, more likely to have hypertension, DM and prior CHD as well as having a poorer NT ProBNP, troponin, lipid profile and kidney function (Supplementary Table S1).

**Table 1:** Baseline characteristics.

	All (*n* = 922)	Men (*n* = 717)	Women (*n* = 205)	*p* (men vs. women)
Demographics				
Age, years	69.7 ± 6.2	69.9 ± 6.1	69.0 ± 6.5	0.085
Ethnicity, *n* (%)				<0.0001
Europeans	440 (47.7)	341 (47.6)	99 (48.3)	
South Asians	335 (36.3)	291 (40.6)	44 (21.5)	
African Caribbean	147 (16)	85 (11.8)	62 (30.2)	
Height, cm	168.3 ± 8.3	171.0 ± 6.8	158.9 ± 6.0	<0.0001
Body mass index, kg/m^2^	26.3 ± 3.5	26.2 ± 3.3	26.8 ± 4.2	0.038
Waist: hip ratio	0.97 ± 0.07	0.99 ± 0.06	0.90 ± 0.07	<0.0001
Clinical history				
Systolic blood pressure, mmHg	140.6 ± 18.2	141.7 ± 17.8	136.8 ± 18.8	0.001
Diastolic blood pressure, mmHg	76.7 ± 9.6	77.6 ± 9.8	73.9 ± 8.4	<0.0001
Heart rate, bpm	67.3 ± 11.7	66.9 ± 11.9	68.7 ± 10.6	0.039
Hypertension, *n* (%)	583 (63.2)	462 (64.4)	121 (59.0)	0.157
Known diabetes, *n* (%)	242 (26.3)	201 (28.0)	41 (20.0)	0.021
Prior coronary heart disease, *n* (%)	140 (15.2)	125 (17.4)	15 (7.3)	<0.0001
Smoking status, *n* (%) never/ex/current				<0.0001
Never	522 (56.9)	381 (53.5)	141 (68.8)	
Ex.	332 (36.2)	285 (40.0)	47 (22.9)	
Current	63 (6.9)	46 (6.5)	17 (8.3)	
Medications				
Anti-diabetic drugs, *n* (%)	154 (16.7)	130 (18.1)	24 (11.7)	0.030
Lipid lowering drugs, *n* (%)	474 (51.4)	395 (55.1)	79 (38.5)	<0.0001
Laboratory/imaging				
Fasting blood cholesterol: HDL ratio	3.6 ± 0.96	3.6 ± 0.96	3.4 ± 0.93	0.013
Fasting blood triglycerides, mmol/l	1.2 ± 0.63	1.2 ± 0.63	1.1 ± 0.61	0.061
HbA1c, %	6.2 ± 0.91	6.2 ± 0.94	6.1 ± 0.78	0.122
eGFR, ml/min/m^2^	73.5 ± 18.9	72.1 ± 18.5	78.7 ± 19.2	<0.0001
Microalbuminuria, *n* (%)				0.159
ACR < 3 mg/mmol	822 (89.2)	633 (88.3)	189 (92.2)	
ACR ≥ 3 mg/mmol	85 (9.2)	73 (10.2)	12 (5.8)	
Proteinuria, ACR ≥ 70 mg/mmol	15 (1.6)	11 (1.5)	4 (2.0)	
Retinopathy categories				0.208
None	532 (69.1)	415 (67.9)	117 (73.6)	
Mild/non-proliferative	215 (27.9)	175 (28.6)	40 (25.2)	
Moderate/proliferative	23 (3.0)	21 (3.4)	2 (1.3)	
NT ProBNP, pg/ml	85[47–168]	81[47–167]	100.5[53–183]	0.055
Troponin, pg/ml	6.9[4.3–10.7]	7.6[5.1–11.6]	4.5[2.1–6.7]	<0.0001
Conventional echocardiography				
LVIDd, cm/m^2^	2.4 ± 0.25	2.4 ± 0.24	2.5 ± 0.25	0.102
LVIDs, cm/m^2^	1.6 ± 0.26	1.6 ± 0.27	1.6 ± 0.23	0.211
IVSd, cm	1.2 ± 0.21	1.2 ± 0.21	1.1 ± 0.19	0.0002
PWd, cm	1.0 ± 0.16	1.0 ± 0.16	1.0 ± 0.18	0.0004
Relative wall thickness	0.46 ± 0.1	0.46 ± 0.1	0.48 ± 0.1	0.005
EF, %	62.0 ± 9.7	61.4 ± 9.9	64.1 ± 8.8	0.0002
EDV, ml/m^2^	49.8 ± 10.5	51.0 ± 10.5	45.5 ± 9.4	<0.0001
ESV, ml/m^2^	19.2 ± 7.8	20.0 ± 8.1	16.4 ± 5.4	<0.0001
LV Mass, g/m^2^	93.5 ± 22.6	95.7 ± 22.1	85.8 ± 22.5	<0.0001
LV Mass, g/m^2.7^	42.4 ± 11.3	42.6 ± 11.0	41.9 ± 12.3	0.493
LV remodeling (2DE)				0.003
Normal	265 (28.7)	218 (30.4)	47 (22.9)	
Concentric remodeling	478 (51.8)	377 (52.6)	101 (49.3)	
Concentric hypertrophy	151 (16.4)	101 (14.1)	50 (24.4)	
Eccentric hypertrophy	28 (3.1)	21 (2.9)	7 (3.4)	
LA diameter, cm/m^2.7^	1.1 ± 0.15	1.1 ± 0.14	1.2 ± 0.15	<0.0001
E wave, cm/sec	62.9 ± 16.1	62.6 ± 16.4	64.1 ± 14.9	0.235
A wave, cm/sec	75.8 ± 17.8	75.3 ± 17.6	77.2 ± 18.3	0.197
Deceleration time, msec	241.9 ± 50.1	243.4 ± 51.2	236.6 ± 45.6	0.071
E/A ratio	0.85 ± 0.23	0.85 ± 0.23	0.86 ± 0.23	0.778
e′, cm/s	7.1 ± 1.8	7.1 ± 1.8	6.8 ± 1.6	0.004
a′, cm/s	10.1 ± 1.8	10.2 ± 1.8	9.6 ± 1.7	<0.0001
s′, cm/s	7.4 ± 1.4	7.6 ± 1.4	7.0 ± 1.2	<0.0001
E/e′	9.4 ± 3.1	9.2 ± 3.1	9.9 ± 3.2	0.006
Ed (LV diastolic stiffness)	0.11 ± 0.05	0.10 ± 0.04	0.14 ± 0.05	<0.0001
3D echocardiography				
EF, %	61 ± 6.8	60.3 ± 6.9	63.5 ± 5.6	<0.0001
EF < 50%. *n* (%)	42 (4.6)	41 (5.7)	1 (0.5)	0.002
EDV, ml/m^2^	43.8 ± 9.6	44.8 ± 9.9	40.1 ± 7.3	<0.0001
ESV, ml/m^2^	17.2 ± 5.8	18.0 ± 6.1	14.7 ± 3.7	<0.0001
LVRI, g/ml	1.5 ± 0.37	1.5 ± 0.37	1.5 ± 0.34	0.539
LVSI	0.33 ± 0.09	0.33 ± 0.09	0.34 ± 0.09	0.282
LV Mass, g/m^2^	64.6 ± 13.2	66.1 ± 13.3	58.9 ± 11.1	<0.0001
LV Mass, g/m^2.7^	29.2 ± 6.3	29.4 ± 6.4	28.6 ± 6.0	0.170
SV, ml/m^2^	26.5 ± 5.7	26.8 ± 5.9	25.4 ± 5.0	0.0005
CO, l/min	3.3 ± 0.85	3.4 ± 0.87	2.9 ± 0.69	<0.0001
LV remodeling (3DE)				<0.0001
Normal	457 (49.6)	353 (49.2)	104 (50.7)	
concentric remodelling	409 (44.4)	336 (46.9)	73 (35.6)	
concentric hypertrophy	52 (5.6)	26 (3.6)	26 (12.7)	
eccentric hypertrophy	4 (0.4)	2 (0.3)	2 (1.0)	
Ed (LV diastolic stiffness)	0.12 ± 0.05	0.12 ± 0.05	0.15 ± 0.06	<0.0001
Vascular measures				
2DE TAC, ml/mmHg.m^2^	0.50 ± 0.16	0.51 ± 0.16	0.49 ± 0.16	0.125
2DE TAC, ml/mm Hg	0.92 ± 0.32	0.96 ± 0.32	0.83 ± 0.29	<0.0001
3DE TAC, ml/mmHg.m^2^	0.44 ± 0.13	0.44 ± 0.13	0.43 ± 0.13	0.297
3DE TAC, ml/ mmHg	0.81 ± 0.27	0.83 ± 0.27	0.73 ± 0.25	<0.0001
2DE Ea, mmHg.m^2^/ml	4.4 ± 0.1.2	4.3 ± 1.2	4.5 ± 1.3	0.115
2DE Ea, mmHg/ml	2.4 ± 0.71	2.3 ± 0.66	2.7 ± 0.81	<0.0001
3DE Ea, mmHg.m^2^/ml	5.0 ± 1.3	4.9 ± 1.3	5.0 ± 1.2	0.571
3DE Ea, mmHg/ml	2.7 ± 0.78	2.7 ± 0.75	3.0 ± 0.81	<0.0001
Central AI, (%)	31.0 ± 9.7	29.9 ± 9.6	34.7 ± 9.0	<0.0001
PWV^a^, M/S	11.3 ± 3.6	11.4 ± 3.4	10.9 ± 4.0	0.132

Data are mean ± SD[or median(interquartile range)] or *n* (%). AI, augmentation index; ACR, albumin: creatinine ratio; CO, cardiac output; Ea, effective arterial elastance; EDV, end-diastolic volume; EF, ejection fraction; eGFR, estimated glomerular filtration rate; ESV, end-systolic volume; HDL, high-density lipoprotein; IVSd, interventricular septal wall thickness in diastole; LA, left atrial; LV, left ventricular; Ed, LV diastolic elastance; LVIDd, LV internal dimension in diastole; LVIDs, LV internal dimension in systole; LVRI, LV remodeling index; LVSI, LV sphericity index; PWd, posterior wall thickness in diastole; SV, stroke volume; TAC, total arterial compliance; 3DE, 3D echocardiography; and 2DE, 2D echocardiography.

^a^ *n* = 701 (*n* = 544 in men, and *n* = 157 in women).

### Outcomes

During follow-up (median, 8 years; range 1–10 years), there were 123 deaths from all-causes (105 in men and 18 in women; incidence rate = 0.017, 0.019 and 0.011 per person-years for the overall population, men and women respectively). During follow-up (median, 7 years; range 0.5–9 years), there were 151 composite cardiovascular endpoints (128 in men and 23 in women; incidence rate = 0.024, 0.026 and 0.016 per person-years for the overall population, men and women respectively).

### 3D LV structural/functional measures and long-term outcomes in sex-pooled analyses

#### All-cause mortality

Lower EF, and higher LV volumes (EDV and ESV) and LVSI were associated with increased risk of all-cause mortality (Table 2). There was no convincing evidence of associations of LVRI, LV mass or SV with increased risk of all-cause mortality. These findings remained essentially unaltered after adjustment for potential confounders (model-1 and model-2). Further adjustment for prior CHD had little or no influence on estimates (Supplementary Table S2).

**Table 2:** Associations between 3DE LV measures and outcomes in the overall population (imputed data).

	Unadjusted	Model-1	Model-2
	Standardized HR (95% CI), *p*-value	Standardized HR (95% CI), *p*-value	Standardized HR (95% CI), *p*-value
**All-cause mortality**			
EF	**0.80 (0.68, 0.94), 0.008**	**0.81 (0.69, 0.97), 0.019**	**0.81 (0.68, 0.96), 0.017**
EDV	**1.19** (1.0, 1.40), 0.039*	**1.21** (1.02, 1.42), 0.025*	**1.20** (1.02, 1.42), 0.030*
ESV	**1.26** (1.06, 1.49), 0.010*	**1.27** (1.06, 1.52), 0.009*	**1.26** (1.06, 1.51), 0.010*
SV	1.03 (0.87, 1.23), 0.717	1.06 (0.90, 1.26), 0.486	1.06 (0.89, 1.26), 0.514
LVRI^a^	0.92 (0.77, 1.11), 0.410*	0.87 (0.72, 1.05), 0.146*	0.86 (0.71, 1.03), 0.096*
LVSI^a^	**1.18** (1.0, 1.40), 0.048*	1.16 (0.97, 1.39), 0.100*	1.15 (0.96, 1.38), 0.137*
LV mass	1.15 (0.97, 1.36), 0.119	1.12 (0.93, 1.34), 0.234	1.09 (0.91, 1.31), 0.352
*123 deaths for EF and volumes, and 118 for LVRI, LVSI and LV mass.*			
**Composite cardiovascular endpoint**			
EF	0.90 (0.77, 1.04), 0.161*	0.91 (0.78, 1.06), 0.240*	0.92 (0.79, 1.07), 0.273*
EDV	**1.28** **(1.11, 1.48), 0.001**	**1.27** **(1.10, 1.47), 0.002**	**1.22** **(1.05, 1.42), 0.008**
ESV	**1.24** (1.06, 1.45), 0.007*	**1.23** (1.05, 1.45), 0.011*	**1.20** (1.02, 1.40), 0.028*
SV	**1.18** **(1.02, 1.38), 0.030**	**1.18** **(1.02, 1.38), 0.031**	**1.14** **(0.98, 1.33), 0.085**
LVRI	0.88 (0.74, 1.05), 0.156*	0.90 (0.76, 1.07), 0.238*	0.90 (0.76, 1.08), 0.253*
LVSI	1.14 (0.98, 1.33), 0.088*	1.11 (0.95, 1.30), 0.196*	1.10 (0.94, 1.29), 0.230*
LV mass	1.16 (0.99, 1.36), 0.065	**1.19** **(1.02, 1.39), 0.031**	1.16 (0.99, 1.36), 0.071
*151 composite cardiovascular endpoints for EF and volumes, and 142 for LVRI, LVSI and LV mass.*			

*N* = 922 for EF and volumes. *N* = 891 for LVRI, LVSI and LV mass. Model-1: adjusted for age, sex and ethnicity. Model 2: model-1 + systolic blood pressure, antihypertensive medication, cholesterol:HDL ratio, body mass index, diabetes mellitus, and smoking. ESV is log transformed. HR, hazard ratio; and SD, standard deviation. Remaining abbreviations as in Table 1. EF, SD = 6.8%; EDV, SD = 9.6 ml/m^2^; ESV, SD = 5.8 ml/m^2^; SV, SD = 5.7 ml/m^2^; LVRI, SD = 0.37 g/ml; LVSI, SD = 0.086; and LV mass, SD = 13.2 g/m^2^.

Values in bold indicate statistical significance.

^a^ The lower numbers are due to missing LV mass measures.

* *P* (interaction) <0.1.

#### Composite cardiovascular endpoint

Higher LV volumes (EDV, ESV and SV) were associated with an increased risk of composite cardiovascular endpoint (Table 2). There was no convincing evidence of an association between EF and LVRI with the composite cardiovascular endpoint. While estimates suggested that higher LVSI and LV mass were associated with increased risk of the composite cardiovascular endpoint, the precision of the estimates was wide. Further adjustment for prior CHD had little/no influence or slightly attenuated the associations (Supplementary Table S2).

There was no evidence that prior CHD modified the associations between outcomes and 3DE LV measures. Complete case analysis models were similar to the primary models with imputation (Supplementary Table S3). Accounting for deaths from non-cardiovascular cause as a competing risk (Supplementary Table S4) had minimal effects on associations, and excluding individuals with prior CHD (Supplementary Table S5) also had little influence except that the precision of estimates was decreased due to reduced sample size and total number of events.

### Sex as a modifier of associations between LV structural/functional measures and long-term outcomes

#### All-cause mortality

**Figure 1:**
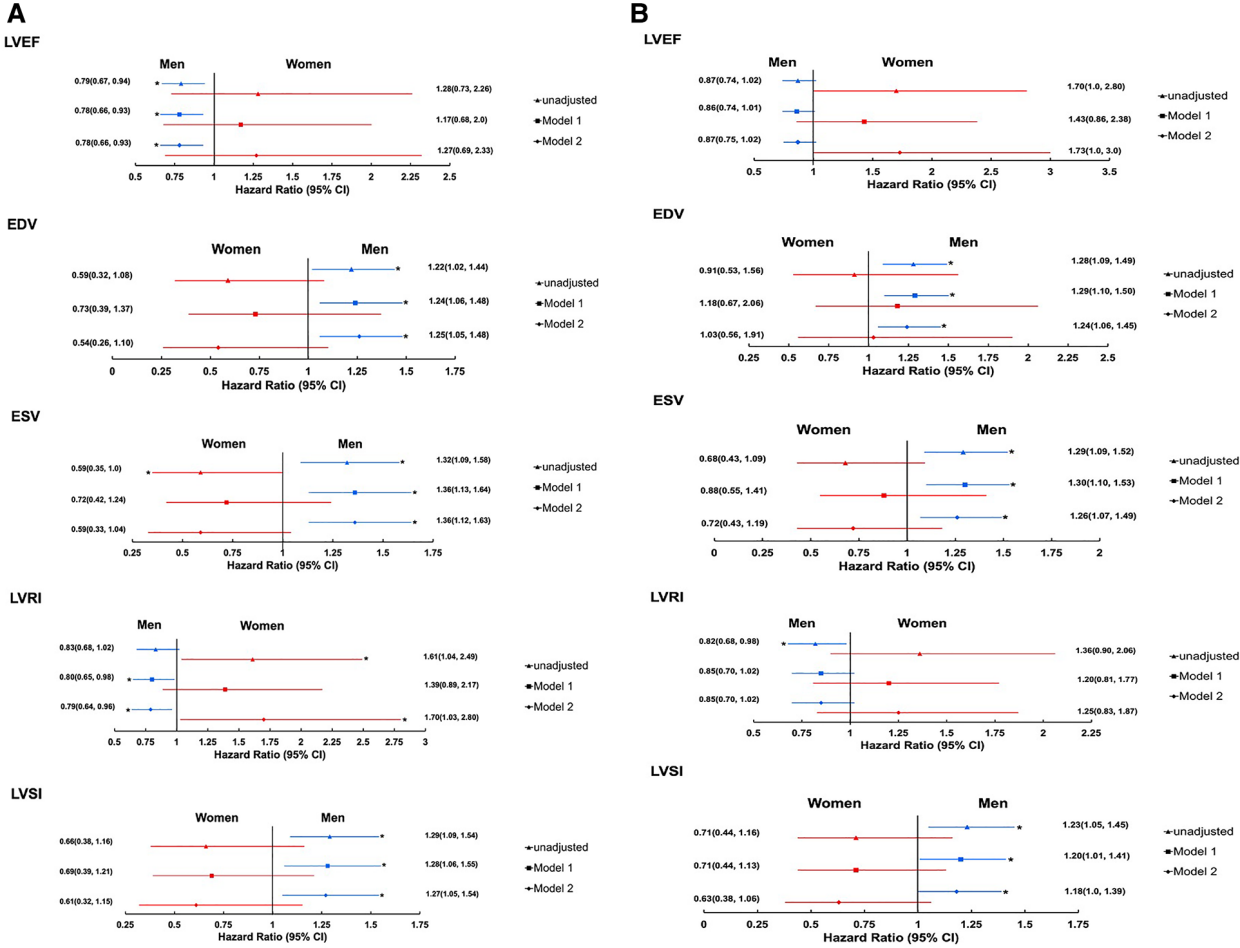
Forest plots showing the associations between 3DE LV measures, and all-cause mortality (**A**) and composite cardiovascular endpoint (**B**) stratified by sex (imputed data). Estimates are HR(95% CI) per SD change. Model-1: adjusted for age and ethnicity. Model-2: model-1 + systolic blood pressure, antihypertensive medication, cholesterol:HDL ratio, body mass index, diabetes mellitus, and smoking. 3D ESV is log transformed. Men: *N* = 717(128 composite cardiovascular endpoints and 105 deaths of all-cause) for EF, EDV, and ESV; *N* = 699(121 composite cardiovascular endpoints and 101 death of all-causes) for LVRI and LVSI (EF, SD = 6.9%; EDV, SD = 9.9 ml/m^2^; ESV, SD = 6.1 ml/m^2^; LVRI, SD = 0.37 g/ml; and LVSI, SD = 0.086). Women: *N* = 205(23 composite cardiovascular endpoints and 18 deaths of all-cause) for EF, EDV, and ESV; *N* = 192(21 composite cardiovascular endpoints and 17 deaths of all-cause) for LVRI and LVSI (EF, SD = 5.6%; EDV, SD = 7.3 ml/m^2^; ESV, SD = 3.7 ml/m^2^; LVRI, SD = 0.34 g/ml; and LVSI, SD = 0.087). *Indicates *p* < 0.05. Abbreviations are as in Table 2.

Sex modified the associations between several 3DE LV structural measures and all-cause mortality (*p*-interaction: EDV = 0.023, ESV = 0.004, LVSI = 0.023 and LVRI = 0.006). However, evidence that sex modified the association between EF and all-cause mortality was unconvincing (*p*-interaction: EF = 0.103). Higher LV volumes (EDV and ESV) and LVSI and lower EF and LVRI were associated with increased risk of all-cause mortality in men but not women (Figure 1A). By contrast, associations between LV volumes (EDV and ESV), LVRI and, to a lesser extent, EF and LVSI and all-cause mortality were reversed in women (Figure 1A), such that lower volumes (EDV and ESV), and higher LVRI were associated with higher risk of all-cause mortality in women.

Sex did not modify the associations between SV and LV mass, and all-cause mortality (*p* interaction: SV = 0.139 and LV mass = 0.965, data not shown).

#### Composite cardiovascular endpoint

Sex modified the associations between several 3DE LV measures and the composite cardiovascular endpoint except for EDV (*p* interaction: EF = 0.016, EDV = 0.0228, ESV = 0.011, LVRI = 0.024, and LVSI = 0.034). Increased LV volumes (EDV and ESV) and LVSI, and decreased EF and LVRI were associated with increased risk of composite cardiovascular endpoint in men (Figure 1B) but not in women. There was weak evidence that an increase in EF was independently associated with increased risk of composite cardiovascular endpoint [HR(95% CI) per SD change, 1.73 (1.0, 3.0); Figure 1B]. Accounting for deaths from non-cardiovascular cause as a competing risk (Supplementary Table S4) had minimal effects on associations.

Sex did not modify the associations between SV and LV mass and composite cardiovascular endpoint (*p* interaction: SV = 0.850 and LV mass = 0.580; data not shown).

Complete case analysis models for sex-stratified data showed minimal differences from the primary models with imputation (Supplementary Tables S6, S7), and excluding individuals with prior CHD also had little influence except that the precision of estimates was decreased due to reduced sample size and total number of events(Supplementary Tables S8, S9). Nelson-Aalen cumulative hazard curves for outcomes are shown in the data supplement (Supplementary Figure S3).

### Potential mediators of sex differences

#### LV diastolic function, macro and microvascular disease

**Figure 2:**
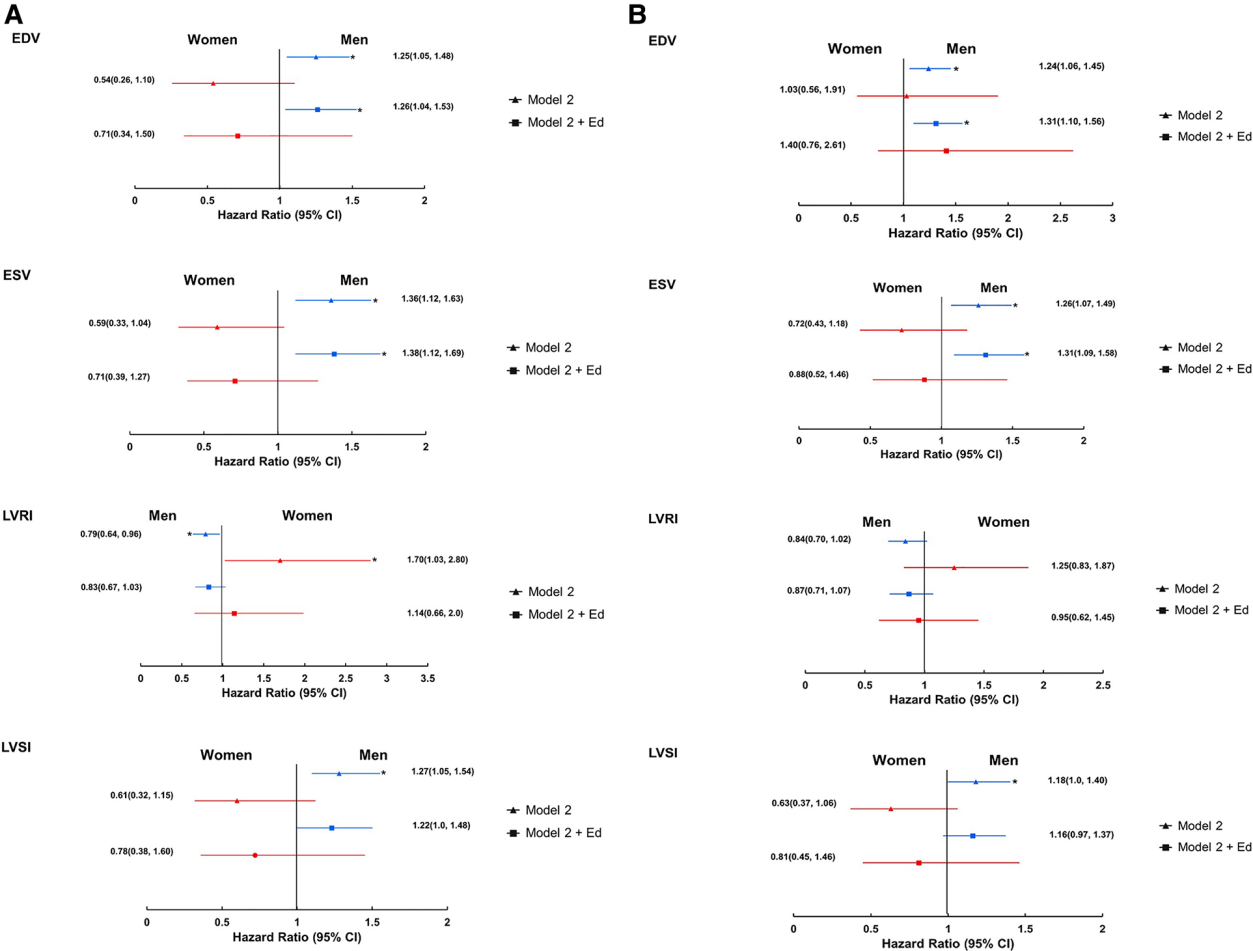
Forest plots showing the associations between 3DE LV measures and all-cause mortality (**A**) and composite cardiovascular endpoint (**B**) stratified by sex; role of diastolic function (imputed data). *Indicates *p* < 0.05. Ed, LV diastolic stiffness. Remaining abbreviations as in Table 2. Footnotes as in Figure 1.

Adjustment for Ed, a marker of LV diastolic stiffness, attenuated the associations between LV structural measures and all-cause mortality in women, but not in men resulting in slightly smaller sex differences (Figure 2A). Adjustment for E/e′ had negligible effects (Supplementary Table S10). Findings were similar for associations with the composite cardiovascular endpoint (Figure 2B and Supplementary Table S10).

**Figure 3:**
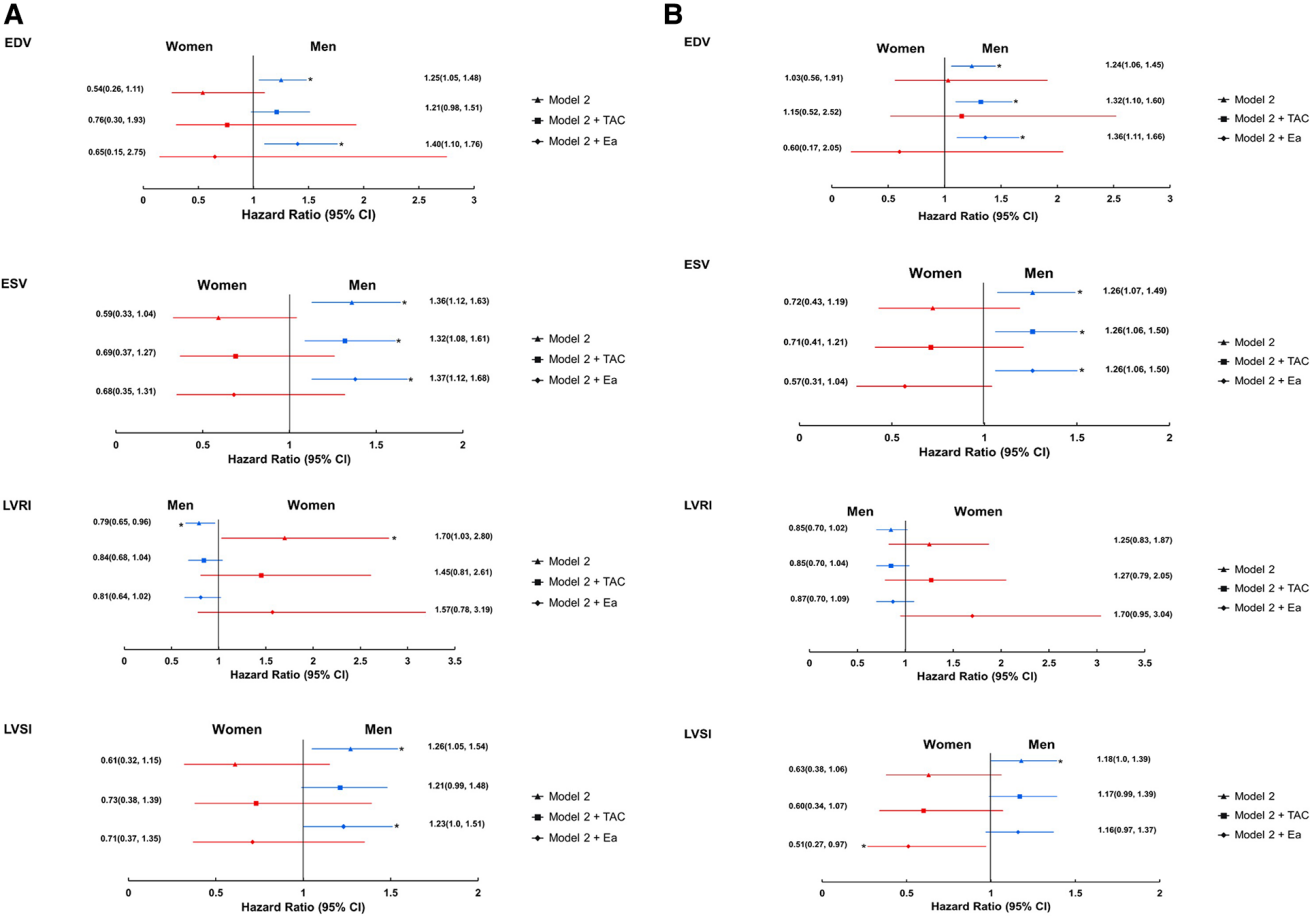
Forest plots showing the associations between 3DE-derived LV measures and all-cause mortality (**A**) and composite cardiovascular endpoint (**B**) stratified by sex; role of macro-vascular disease (imputed data). *Indicates *p* < 0.05. Ea, effective arterial elastance; and TAC, total arterial compliance. Remaining abbreviations as in Table 2. Footnotes as in Figure 1.

Adjustment for TAC and Ea, markers of arterial stiffness, attenuated the associations between 3D LV structural measures and all-cause mortality in women, but not in men, resulting in smaller sex differences (Figure 3A). Results remained similar when indexed TAC and Ea were used (Supplementary Table S10). Adjustment for AI and PWV had negligible effects (Supplementary Table S10). There was no clear evidence that adjustment for macrovascular disease influenced sex differences in associations with the composite cardiovascular endpoint (Figure 3B and Supplementary Table S10).

**Figure 4:**
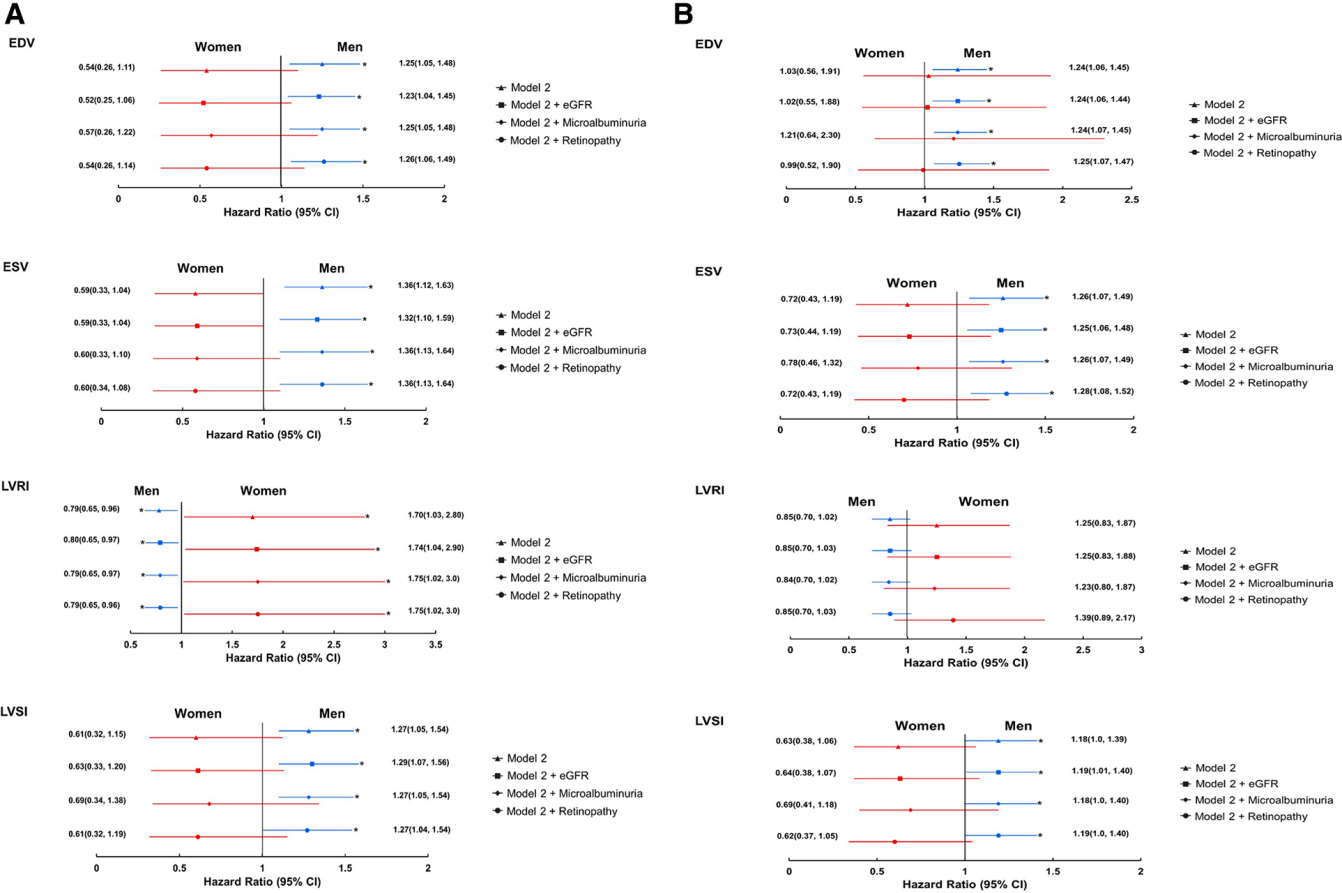
Forest plots showing the associations between 3DE-derived LV measures and all-cause mortality (**A**) and composite cardiovascular endpoint (**B**) stratified by sex; role of micro-vascular disease(imputed data). *Indicates *p* < 0.05. eGFR, estimated glomerular filtration rate. Remaining abbreviations as in Table 2. Footnotes as in Figure 1.

Adjustment for markers of microvascular disease had negligible effect on associations between LV structural measures and all-cause mortality, and between LV structural measures and EF, and composite cardiovascular endpoint in either sex (Figure 4 and Supplementary Supplementary Table S10).

All complete case analyses were similar to imputed analysis (Supplementary Tables S11–S13).

## Discussion

3DE imaging is currently recommended as a method of choice for assessing LV structure and function. Despite this, there is limited evidence regarding its usefulness in the general population. Our study provides important insights regarding the long-term prognostic utility of 3DE in this setting and identifies potentially important sex-differences in some associations with outcomes which were only partially explained by differences in LV stiffness and macrovascular disease.

In our study, lower EF and higher LV volumes by 3DE were independently associated with increased risk of all-cause mortality. Higher LV volumes and LV mass also predicted cardiovascular morbidity. Evidence from previous studies reporting the prognostic value of 3DE has been limited to selected high risk patients (3–5). A small study by Caselli et al. reported associations between composite cardiovascular events (MI, stroke, or cardiovascular death) and 3DE EF, LV volumes and mass in a population of 178 consecutive outpatients over 45 months, but failed to account for differences in patients' risk profile (5). Stanton et al. found in a retrospective study of consecutive 455 patients that 3DE EF, EDV and ESV were all independent predictors of a composite endpoint of all-cause mortality and cardiac hospitalization over a mean follow-up of 6.6 years (4). Another recent retrospective study of hospital inpatients by Medvedofsky et al. reported an association between 3DE EF and cardiovascular mortality in 416 inpatients over a mean follow-up of 5 years (3). These studies of selected clinical samples employed limited adjustment for potential confounders leaving them subject to potential biases; but are broadly consistent with our findings.

LV function and shape are interrelated, but LV shape is not routinely assessed in clinical practice. In the pooled analysis of this study, 3DE LVSI and LVRI were observed to predict all-cause mortality. In the Multi-Ethnic Study of Atherosclerosis (MESA) study, LV shape/geometry indices, derived from cardiac magnetic resonance imaging (CMR), were strong independent predictors of cardiovascular events in asymptomatic community-dwelling individuals free of known cardiovascular disease (23); however, using CMR as a screening tool in large populations might not be cost-effective (23). Potentially, therefore, 3DE LV shape analysis combined with conventional indices of LV function (EF) and structure (volumes and mass) may provide more complete description of the LV, providing additional diagnostic and/or long-term prognostic information.

### Sex as a modifier of associations between 3 days LV structural/functional measures and long-term outcomes

Cardiovascular aging differs by sex, women typically have smaller cavity volumes and mass, and higher EF and mass to volume ratio and higher arterial stiffness than men (6, 8). The greater degree of concentric remodeling in women may be linked to the greater prevalence of diastolic HF in women (7). In keeping with these known differences, we found that sex modified associations between 3DE indices and outcomes. Decreased 3D LVRI (and EF) and increased volumes (EDV and ESV) and LVSI were associated with increased risk of mortality (and morbidity) more strongly in men, while associations were null or reversed in women. Interestingly, higher EF was observed to be associated with increased risk of morbidity in women independent of other risk factors. This is consistent with previous evidence of “supra-EF” as a compensatory mechanism associated with concentric remodeling in women (i.e., smaller cavity volumes with greater contraction) (23). Adjustment for Ed, Ea and TAC attenuated the associations between 3DE indices of LV structure and the long-term risk of mortality in women and reduced the differences in strength of association between women and men. It seems possible therefore that sex-differences in associations between LV structure and mortality risk may be partially explained by higher ventricular and arterial stiffness in women. This is consistent with evidence from HF patients, where LV systolic dysfunction and eccentric remodeling/hypertrophy carry prognostic information in men, while impaired diastolic function and LV concentric remodeling/hypertrophy may be more important for risk in women. In contrast, despite microvascular disease also being more common in older women (9), there was no evidence to suggest that microvascular disease contributed to the sex differences observed in this study. A growing body of evidence suggests that sex-differences particularly in ventricular remodeling might be attributable to postmenopausal withdrawal of estrogen (24). Whether this explains the difference observed in our study requires further research but might have potential to open ways to novel treatments and ultimately benefit patients of both sexes.

#### Study strengths and limitations

This study has several limitations. SABRE is a prospective observational population-based tri-ethnic longitudinal study of older people from the UK general population and by design recruited more men than women; therefore, estimates in women, who also experienced fewer events, are less precise. We cannot exclude the possibility that some associations found might have occurred by chance considering the number of analyses (multiple testing). 3DE was not acquired in participants with AF or if the image quality was too poor, and there was a small subset of participants who had attended the clinic before the 3D probe became available (11, 25). These exclusions may have introduced some bias. The study was performed between 2008 and 2011, and while the technology was state-of-the-art at the time, 3DE technology and 3D algorithms have improved subsequently, but the use of technology which becomes outdated is unavoidable in prospective outcome studies with long follow-up. Differences in probe frequency and resolution could contribute to differences between 2DE and 3DE data. The use of diastolic elastance calculated as (E/e′)/EDV as a measure of myocardial stiffness has not been validated in this population, and E/e′ as a measure of filling pressure has limitations, although guidelines recommend it. As in any observational study, residual confounding cannot be fully excluded despite extensive adjustment for potential confounders.

## Conclusions

In a large tri-ethnic sample of the UK general population, altered LV function and structure assessed by 3DE were independently associated with long-term risk of all-cause mortality and cardiovascular morbidity. However, the direction of several of these associations differed by sex, suggesting that sex-differences in LV remodeling patterns may influence mortality and morbidity risk in the general population. Modification of associations by sex seemed to be partially explained by higher ventricular and arterial stiffness in women (26, 27, 28).

## Data Availability

The datasets presented in this article are not readily available for reasons of confidentiality. Requests to access the datasets should be directed to MRCLHA.swiftinfo@ucl.ac.uk with a brief outline of your proposed research and the data that you would need.
