# Supplementary material for: Sex-differences in associations of LV structure and function measured by echocardiography with long-term risk of mortality and cardiovascular morbidity
Source: Front Cardiovasc Med. 2023 Apr 25;10:1144964. doi: 10.3389/fcvm.2023.1144964 (PMC10166834; doi:10.3389/fcvm.2023.1144964)
Supplement: Supplementary file 1 [file Table1.docx]

Supplementary Material

Sex-differences in LV structure and function by 3D echocardiography in associations with long-term risk of mortality and cardiovascular morbidity

Lamia Al Saikhan^1^, Chloe Park^2^, Therese Tillin^2^, Siana Jones^2^, Darrel Francis^3^, Jamil Mayet^3^, Nish Chaturvedi^2^, MD, Alun Hughes^2^

*** Correspondence:** Alun Hughes. alun.hughes@ucl.ac.uk.


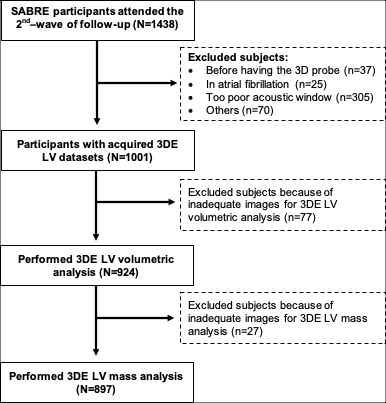
**Supplementary Methods**

Figure-S1 Flow diagram showing the enrollment of SABRE participants in the present study. 3DE, 3D echocardiography; LV, left ventricular.


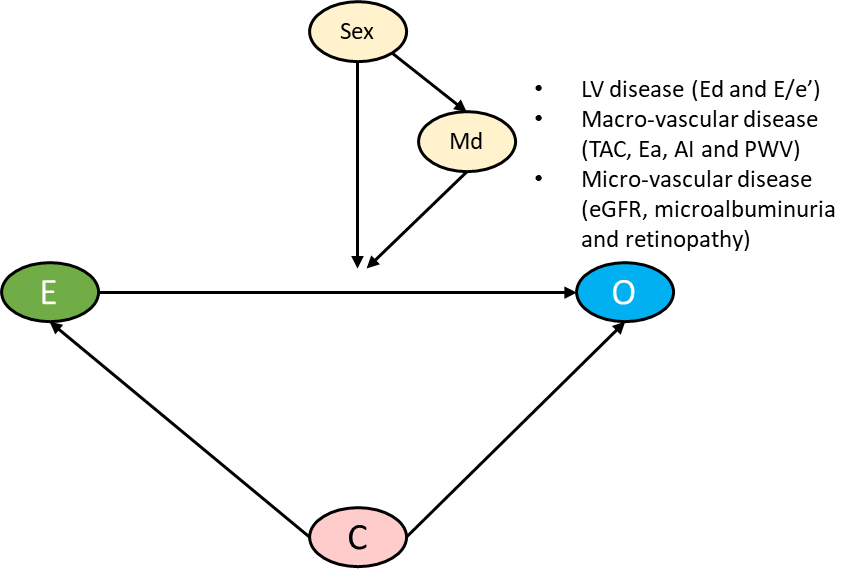


Figure-S2 An illustrative DAG demonstrating the proposed potential mechanisms of the sex-difference in associations between 3DE LV structure (E=exposure) and adverse outcomes (O=outcome). C=confounder; Md=mediator or a factor that thought to explain the sex-difference (i.e. moderation/interaction/effect modification). Abbreviations: AI, augmentation index; Ea, effective arterial elastance; Ed, LV diastolic elastance; eGFR, estimated glomerular filtration rate; LV, left ventricular; PWV, pulse wave velocity; and TAC, total arterial compliance.

**Statistical Analysis (Supplemental Material)**

The number of missing values for covariates ranged from 0 to 221 (23.97%). PWV was the variable with the most missing values (n=221, 23.97%), due to equipment failure followed by retinopathy (n=152, 16.49%), largely due to refusal or contra-indications for pupillary dilation.

**Supplementary Results**


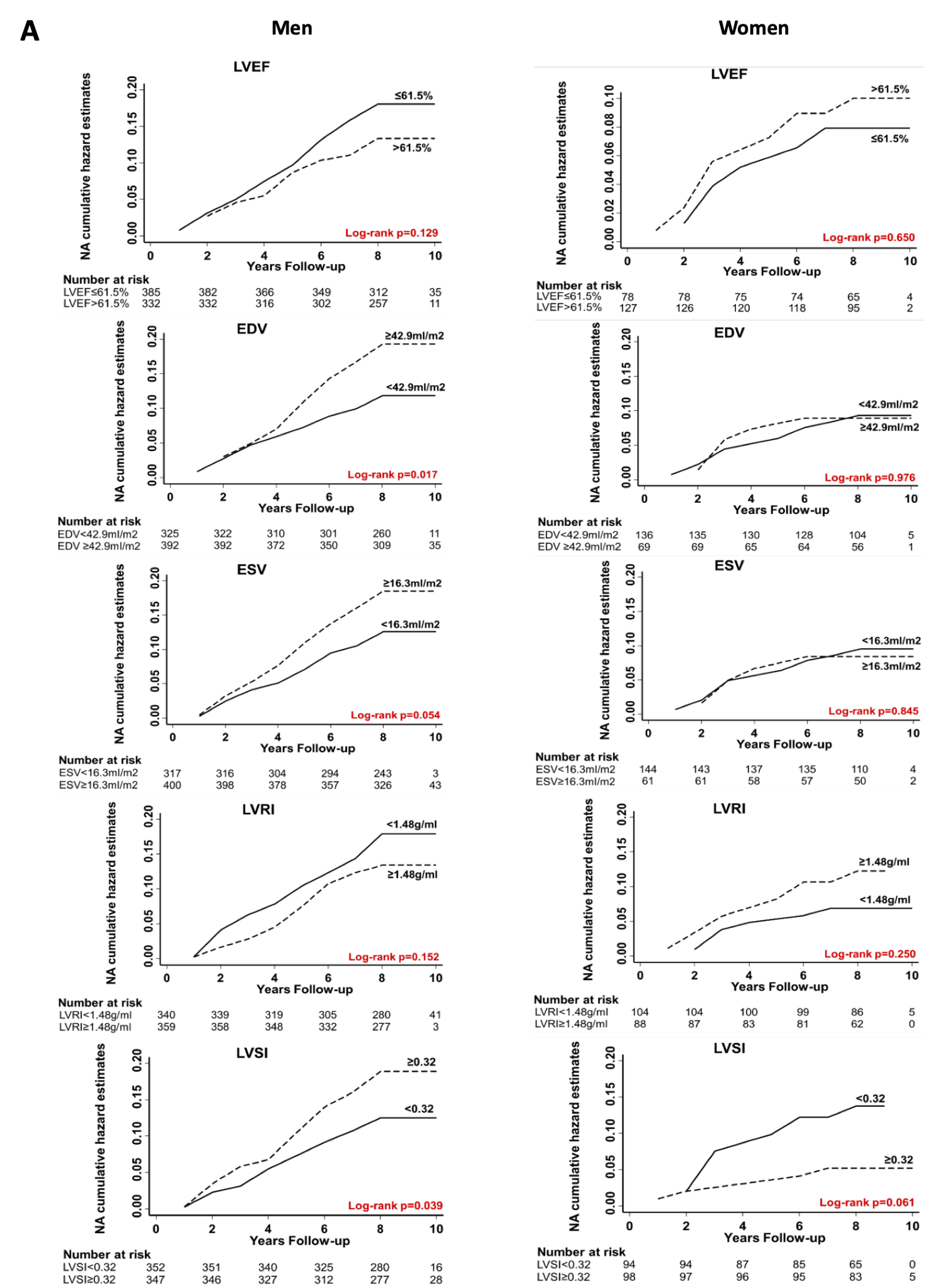


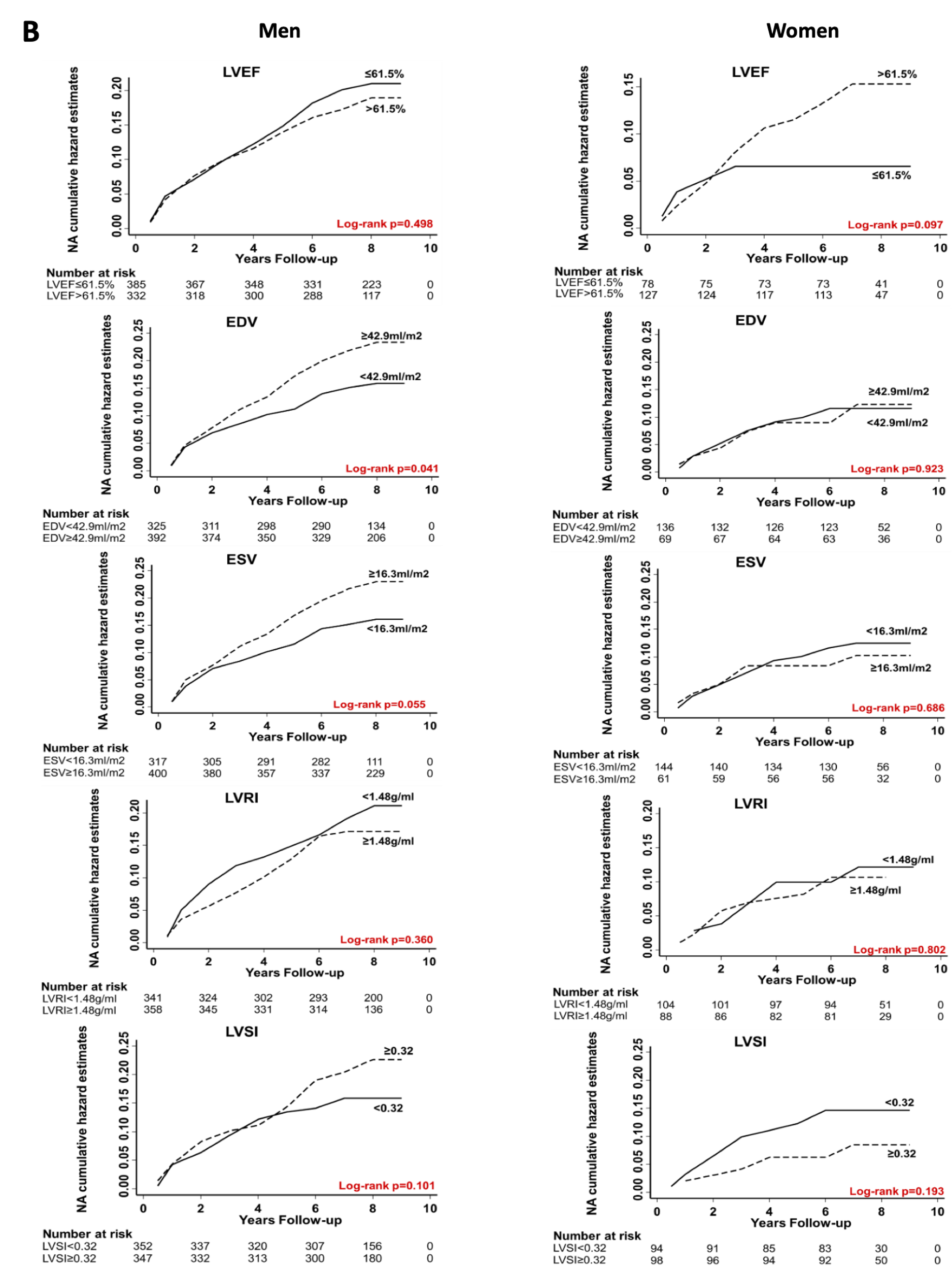
**Figure-S3** Nelson-Aalen cumulative hazard curves by medians for 3D LV measures. All-cause mortality(A) and composite cardiovascular endpoint(B) stratified by sex. Dashed line=≥median, solid line=<median (for EF; Dashed line=>median, solid line=≤median).

| **Table-S1 Baseline clinical characteristics of SABRE participants with and without 3DE** | | | |
| --- | --- | --- | --- |
|  | **+3DE (n=922)** | **-3DE (n=485)** | ***P*** |
| **Demographics** | | | |
| Age, years | 69.7±6.2 | 69.8±6.0 | 0.95 |
| Male, n(%) | 717(77.8) | 352(72.6) | 0.030 |
| Ethnicity, n(%) |  |  | 0.94 |
| Europeans | 440(47.7) | 229(47.2) |  |
| South Asians | 335(36.3) | 175(36.1) |  |
| African Caribbean | 147(16) | 81(16.7) |  |
| Height, cm | 168.3±8.3 | 167.2±9.7 | 0.031 |
| Body mass index, kg/m2 | 26.3±3.5 | 30.0±5.8 | <0.0001 |
| Waist: hip ratio | 0.97±0.07 | 0.99±0.08 | <0.0001 |
| **Clinical history** | | | |
| Systolic blood pressure, mmHg | 140.6±18.2 | 139.2±17.1 | 0.142 |
| Diastolic blood pressure, mmHg | 76.7±9.6 | 77.5±1.0 | 0.149 |
| Heart rate, bpm | 67.3±11.7 | 70.2±13.1 | <0.0001 |
| Hypertension, n(%) | 583(63.2) | 360(74.2) | <0.0001 |
| Known diabetes, n(%) | 242(26.3) | 198(40.8) | <0.0001 |
| Prior coronary heart disease, n(%) | 140(15.2) | 93(19.2) | 0.056 |
| Smoking status, n(%) never/ex/current |  |  | 0.353 |
| Never | 522(56.9) | 274(57.0) |  |
| Ex. | 332(36.2) | 183(38.0) |  |
| Current | 63(6.9) | 24(5.0) |  |
| **Medications** | | | |
| Anti-diabetic drugs, n(%) | 154(16.7) | 148(30.5) | <0.0001 |
| Lipid lowering drugs, n(%) | 474(51.4) | 310(63.9) | <0.0001 |
| **Laboratory/imaging** | | | |
| Fasting blood cholesterol: HDL ratio | 3.6±0.96 | 3.6±1.1 | 0.572 |
| Fasting blood triglycerides, mmol/l | 1.2±0.63 | 1.4±0.72 | <0.0001 |
| HbA1c, % | 6.2±0.91 | 6.6±1.2 | <0.0001 |
| eGFR, ml/min/m2 | 73.5±18.9 | 70.5±20.6 | 0.007 |
| Microalbuminuria, n(%) |  |  | 0.034 |
| ACR<3 mg/mmol | 822(89.2) | 409(84.3) |  |
| ACR≥3 mg/mmol | 85(9.2) | 64(13.2) |  |
| Proteinuria, ACR≥70 mg/mmol | 15(1.6) | 12(2.5) |  |
| Retinopathy categories |  |  | 0.075 |
| None | 532(69.1) | 241(62.4) |  |
| Mild/non-proliferative | 215(27.9) | 130(33.7) |  |
| Moderate/proliferative | 23(3.0) | 153.9) |  |
| NT ProBNP, pg/ml | 85[47-168] | 104[54-223] | 0.003 |
| Troponin, pg/ml | 6.9[4.3-10.7] | 7.7[5.1-12.6] | 0.005 |
| **Outcomes** |  |  |  |
| All-cause mortality, n(%)  *Incidence rate, per person-years* | 123(13.3)  0.017 | 78(16.1)  0.019 | 0.162 |
| Composite cardiovascular endpoints, n(%)  *Incidence rate, per person-years* | 151(16.4)  0.024 | 100(20.6)  0.03 | 0.048 |
| Data are mean±SD (or median[interquartile range]) or n(%). Abbreviations: ACR, albumin: creatinine ratio; eGFR, estimated glomerular filtration rate; HDL, high-density lipoprotein. | | | |

| **Table-S2 Associations between 3DE LV measures and outcomes after further adjustment for prior CHD (imputed data)** | | | |
| --- | --- | --- | --- |
|  | **Overall population(n=922)** | **Men(n=717)** | **Women(n=205)** |
|  | **Standardized HR (95% CI), p value** | **Standardized HR (95% CI), p value** | **Standardized HR (95% CI), p value** |
| **All-cause mortality** | | | |
| EF | 0.85(0.71, 1.01), 0.069 | 0.81(0.68, 0.97), 0.028 | 1.26(0.69, 2.33), 0.441 |
| EDV | 1.16(0.98, 1.37), 0.069^#^ | 1.21(1.02, 1.44), 0.025 | 0.58(0.29, 1.19), 0.138 |
| ESV | 1.20(1.01, 1.44), 0.039^#^ | 1.30(1.08, 1.57), 0.007 | 0.62(0.35, 1.09), 0.101 |
| SV | 1.05(0.89, 1.25), 0.518 |  |  |
| LVRI* | 0.86(0.70, 1.05), 0.139^#^ | 0.79(0.63, 0.98), 0.035 | 1.60(0.90, 2.85), 0.109 |
| LVSI* | 1.16(0.97, 1.40), 0.110^#^ | 1.28(1.05, 1.56), 0.012 | 0.69(0.36, 1.32), 0.267 |
| LV mass | 1.08(0.90, 1.30), 0.385 |  |  |
|  | *123 deaths for EF and volumes, and 118 for LVRI, LVSI and LV mass.* | *105 deaths for EF and volumes, and 101 for LVRI and LVSI.* | *18 deaths for EF and volumes, and 17 for LVRI and LVSI.* |
| **Composite cardiovascular endpoint** | |  |  |
| EF | 0.98(0.84, 1.15), 0.895^#^ | 0.94(0.81, 1.11), 0.521 | 1.73(1.00, 3.0), 0.052 |
| EDV | 1.15(0.99, 1.34), 0.05 | 1.16(1.0, 1.36), 0.049 | 1.03(0.56, 1.89), 0.923 |
| ESV | 1.10(0.94, 1.29), 0.207^#^ | 1.15(0.97, 1.36), 0.098 | 0.72(0.44, 1.19), 0.203 |
| SV | 1.14(0.98, 1.33), 0.086 |  |  |
| LVRI | 0.93(0.79, 1.10), 0.445 | 0.89(0.74, 1.08), 0.261 | 1.24(0.82, 1.87), 0.293 |
| LVSI | 1.08(0.93, 1.27), 0.310^#^ | 1.16(0.98, 1.37), 0.075 | 0.63(0.37, 1.07), 0.085 |
| LV mass | 1.13(0.97, 1.31), 0.111 |  |  |
|  | *151 composite cardiovascular endpoints for EF and volumes, and 142 for LVRI, LVSI and LV mass.* | *128 composite cardiovascular endpoints for EF and volumes, and 121 for LVRI and LVSI.* | *23 composite cardiovascular endpoints for EF and volumes, and 21 for LVRI and LVSI.* |
| Adjusted for age, sex, ethnicity, systolic blood pressure, antihypertensive medication, cholesterol: HDL ratio, body mass index, diabetes mellitus, smoking, and history of coronary heart disease. ESV is log transformed. EF, SD=6.8%; EDV, SD=9.6ml/m^2^; ESV, SD=5.8ml/m^2^; SV, SD=5.7ml/m^2^; LVRI, SD=0.37g/ml; LVSI, SD=0.086; and LV mass, SD=13.2g/m^2^. Men: EF, SD=6.9%; EDV, SD=9.9ml/m^2^; ESV, SD=6.1ml/m^2^; LVRI, SD=0.37g/ml; and LVSI, SD=0.086. Women: EF, SD=5.6%; EDV, SD=7.3ml/m^2^; ESV, SD=3.7ml/m^2^; LVRI, SD=0.34g/ml; and LVSI, SD=0.087. *The lower numbers are due to missing LV mass measures (N=891; men, N=699; women, N=192). ^#^P(interaction) <0.1. Abbreviations: EDV, end-diastolic volumes; ESV, end-systolic volumes; HR, hazard ratio; LV, left ventricular; EF, ejection fraction; LVRI, LV remodeling index; LVSI, LV sphericity index; SD, standard deviation; and SV, stroke volume. | | | |

| **Table-S3 Associations between 3DE LV measures and outcomes in the overall population (complete case analysis)** | | | | |
| --- | --- | --- | --- | --- |
|  | **Unadjusted** | **Model-1** | **Model-2** | **Model-3** |
|  | **Standardized HR (95% CI), p value** | **Standardized HR (95% CI), p value** | **Standardized HR (95% CI), p value** | **Standardized HR (95% CI), p value** |
| **All-cause mortality** | | | | |
| EF | 0.79(0.67, 0.93), 0.006 | 0.81(0.68, 0.96), 0.016 | 0.81(0.68, 0.96), 0.014 | 0.84(0.71, 1.00), 0.053 |
| EDV | 1.20(1.02, 1.42), 0.029 | 1.21(1.03, 1.43), 0.024 | 1.20(1.02, 1.42), 0.029 | 1.17(1.00, 1.38), 0.063 |
| ESV | 1.27(1.07, 1.51), 0.007 | 1.28(1.07, 1.53), 0.008 | 1.27(1.06, 1.52), 0.009 | 1.22(1.02, 1.46), 0.032 |
| SV | 1.04(0.87, 1.24), 0.675 | 1.06(0.89, 1.26), 0.497 | 1.06(0.89, 1.26), 0.539 | 1.06(0.89, 1.26), 0.527 |
| LVRI | 0.91(0.75, 1.10), 0.336 | 0.86(0.71, 1.03), 0.106 | 0.84(0.70, 1.01), 0.069 | 0.85(0.71, 1.03), 0.099 |
| LVSI | 1.20(1.02, 1.42), 0.033 | 1.18(0.99, 1.42), 0.072 | 1.17(0.97, 1.40), 0.098 | 1.18(0.98, 1.41), 0.077 |
| LV mass | 1.14(0.96, 1.36), 0.141 | 1.10(0.91, 1.32), 0.330 | 1.07(0.89, 1.29), 0.493 | 1.06(0.88, 1.28), 0.515 |
| **Composite cardiovascular endpoint** | |  |  |  |
| EF | 0.90(0.77, 1.05), 0.164 | 0.92(0.78, 1.07), 0.273 | 0.92(0.79, 1.08), 0.307 | 0.99(0.85, 1.15), 0.936 |
| EDV | 1.28(1.10, 1.48), 0.001 | 1.26(1.09, 1.46), 0.002 | 1.21(1.04, 1.41), 0.011 | 1.15(0.99, 1.33), 0.056 |
| ESV | 1.24(1.06, 1.45), 0.008 | 1.22(1.04, 1.43), 0.015 | 1.18(1.01, 1.39), 0.039 | 1.10(0.94, 1.28), 0.232 |
| SV | 1.18(1.02, 1.38), 0.033 | 1.18(1.01, 1.37), 0.038 | 1.14(0.97, 1.33), 0.103 | 1.14(0.97, 1.32), 0.090 |
| LVRI | 0.87(0.74, 1.04), 0.133 | 0.90(0.76, 1.06), 0.207 | 0.90(0.76, 1.07), 0.238 | 0.92(0.78, 1.09), 0.382 |
| LVSI | 1.15(0.98, 1.34), 0.082 | 1.11(0.95, 1.31), 0.190 | 1.10(0.94, 1.29), 0.228 | 1.10(0.93, 1.27), 0.280 |
| LV mass | 1.15(0.98, 1.34), 0.094 | 1.16(0.99, 1.36), 0.059 | 1.14(0.97, 1.33), 0.118 | 1.11(0.95, 1.29), 0.176 |
| N=910 (150 composite cardiovascular endpoints and 120 deaths of all-cause) for EF and volumes. N=879 (141 composite cardiovascular endpoints and 115 deaths of all-cause) for LVRI, LVSI and LV mass. Model-1: adjusted for age, sex and ethnicity. Model-2: model-1 + systolic blood pressure, antihypertensive medication, cholesterol: HDL ratio, body mass index, diabetes mellitus, and smoking. Model-3: model-2 + history of coronary heart disease. ESV is log transformed. Abbreviations: EDV, end-diastolic volumes; ESV, end-systolic volumes; HR, hazard ratio; LV, left ventricular; EF, ejection fraction; LVRI, LV remodelling index; LVSI, LV sphericity index; SD, standard deviation; and SV, stroke volume. EF, SD=6.8%; EDV, SD=9.6ml/m^2^; ESV, SD=5.8ml/m^2^; SV, SD=5.7ml/m^2^; LVRI, SD=0.37g/ml; LVSI, SD=0.086; and LV mass, SD=13.2g/m^2^. | | | | |

| Table-S4 Associations between 3DE LV measures and composite cardiovascular endpoint (complete case analysis; competing risk analysis) | | | | |
| --- | --- | --- | --- | --- |
|  | **Standardized Sub-HR (95% CI), p value** | | | |
|  | **Unadjusted** | **Model-1** | **Model-2** | **Model-3** |
| **Overall population** | | |  |  |
| EF | 0.90(0.76, 1.05), 0.181 | 0.92(0.78, 1.07), 0.282 | 0.92(0.79, 1.08), 0.314 | 0.99(0.85, 1.16), 0.935 |
| EDV | 1.28(1.10, 1.48), 0.001 | 1.26(1.09, 1.46), 0.002 | 1.21(1.04, 1.41), 0.009 | 1.15(1.0, 1.33), 0.047 |
| ESV | 1.24(1.05, 1.46), 0.011 | 1.22(1.04, 1.43), 0.017 | 1.18(1.01, 1.39), 0.038 | 1.10(0.94, 1.28), 0.224 |
| SV | 1.18(1.01, 1.38), 0.033 | 1.18(1.01, 1.37), 0.039 | 1.14(0.97, 1.33), 0.104 | 1.14(0.98, 1.33), 0.091 |
| LVRI | 0.87(0.74, 1.04), 0.120 | 0.90(0.76, 1.05), 0.186 | 0.90(0.76, 1.06), 0.221 | 0.92(0.79, 1.09), 0.347 |
| LVSI | 1.15(0.99, 1.33), 0.061 | 1.11(0.96, 1.29), 0.160 | 1.10(0.95, 1.28), 0.204 | 1.09(0.94, 1.27), 0.266 |
| LV mass | 1.14(0.97, 1.35), 0.113 | 1.16(0.99, 1.36), 0.054 | 1.14(0.97, 1.32), 0.104 | 1.11(0.96, 1.27), 0.131 |
| N=50 competing events (non-CV deaths) for EF and volumes; n=49 for LVRI, LVSI and LV mass. | | | | |
| **Men** | | | | |
| EF 0.87(0.74, 1.02), 0.088 0.87(0.74, 1.02), 0.078 0.88(0.75, 1.02), 0.094 0.95(0.81, 1.11), 0.549  EDV 1.27(1.09, 1.48), 0.002 1.28(1.10, 1.49), 0.001 1.24(1.06, 1.44), 0.005 1.16(1.0, 1.35), 0.040  ESV 1.28(1.08, 1.51), 0.003 1.29(1.09, 1.52), 0.002 1.26(1.07, 1.47), 0.005 1.14(0.97, 1.35), 0.090  LVRI 0.82(0.68, 0.96), 0.017 0.85(0.72, 1.0), 0.051 0.85(0.72, 1.01), 0.060 0.89(0.76, 1.06), 0.196  LVSI 1.23(1.05, 1.42), 0.007 1.19(1.02, 1.39), 0.026 1.18(1.00, 1.38), 0.042 1.16(0.98, 1.36), 0.072  N=46 competing events (non-CV deaths) for EF and volumes; n=45 for LVRI and LVSI. | | | | |
| **Women** | | | | |
| EF 1.77(1.0, 3.1), 0.050 1.53(0.84, 2.78), 0.160 1.81(0.94, 346), 0.075 1.81(0.94, 3.46), 0.074  EDV 0.87(0.50, 1.54), 0.640 1.12(0.58, 2.1), 0.725 0.98(0.49, 1.93), 0.947 0.98(0.49, 1.93), 0.947  ESV 0.64(0.39, 1.05), 0.083 0.82(0.45, 1.50), 0.514 0.68(0.39, 1.20), 0.189 0.68(0.39, 1.20), 0.183  LVRI 1.31(0.79, 2.17), 0.290 1.16(0.71, 1.90), 0.561 1.22(0.76, 1.96), 0.408 1.22(0.76, 1.97), 0.394  LVSI) 0.73(0.46, 1.16), 0.182 0.73(0.47, 1.12), 0.153 0.66(0.40, 1.1), 0.096 0.65(0.39, 1.1), 0.086  N=4 competing events (non-CV deaths). | | | | |
| Overall sample: N=910 (150 composite cardiovascular endpoints) for EF and volumes. N=879 (141 composite cardiovascular endpoints) for LVRI, LVSI and LV mass. Remaining footnotes and abbreviations are as in Table-S3. EF, SD=6.8%; EDV, SD=9.6ml/m^2^; ESV, SD=5.8ml/m^2^; SV, SD=5.8ml/m^2^; LVRI, SD=0.37g/ml; LVSI, SD=0.086; and LV mass, SD=13.2g/m^2^.  Men: N=707 (128 composite cardiovascular endpoints) for EF, EDV, and ESV. N=689 (121 composite cardiovascular endpoints) for LVRI and LVSI. EF, SD=7.0%; EDV, SD=10.0ml/m^2^; ESV, SD=6.1ml/m^2^; LVRI, SD=0.37g/ml; and LVSI, SD=0.086.  Women: N=203 (22 composite cardiovascular endpoints) for EF, EDV, and ESV. N=190 (20 composite cardiovascular endpoints) for LVRI and LVSI. EF, SD=5.6%; EDV, SD=7.3ml/m2; ESV, SD=3.7ml/m2; LVRI, SD=0.34g/ml; and LVSI, SD=0.087. | | | | |

| Table-S5 Associations between 3DE LV measures and outcomes in the overall population without prior CHD (complete case analysis) | | | | | | |
| --- | --- | --- | --- | --- | --- | --- |
|  | **Standardized HR (95% CI), p value** | | | | | |
|  | **Unadjusted** | | | **Model-1** | **Model-2** | |
| **All-cause mortality** | | | | |  | |
| EF | | 0.87(0.70, 1.1), 0.206 | 0.88(0.70, 1.1), 0.298 | | | 0.87(0.69, 1.1), 0.237 |
| EDV | | 1.17(0.94, 1.44), 0.166 | 1.18(0.95, 1.47), 0.126 | | | 1.21(0.97, 1.51), 0.084 |
| ESV | | 1.17(0.94, 1.47), 0.156 | 1.19(0.94, 1.50), 0.145 | | | 1.22(0.97, 1.55), 0.092 |
| SV | | 1.07(0.86, 1.32), 0.542 | 1.09(0.89, 1.35), 0.396 | | | 1.11(0.89, 1.37), 0.327 |
| LVRI | | 0.93(0.74, 1.16), 0.523 | 0.84(0.67, 1.06), 0.140 | | | 0.82(0.66, 1.03), 0.090 |
| LVSI | | 1.20(0.98, 1.47), 0.080 | 1.15(0.93, 1.43), 0.202 | | | 1.13(0.91, 1.42), 0.250 |
| LV mass | | 1.08(0.87, 1.35), 0.472 | 1.0(0.79, 1.26), 0.997 | | | 0.98(0.77, 1.24), 0.858 |
| **Composite cardiovascular endpoint** | | | | |  | |
| EF | 0.92(0.75, 1.1), 0.463 | | | 0.91(0.73, 1.1), 0.381 | 0.91(0.74, 1.1), 0.383 | |
| EDV | 1.20(0.98, 1.47), 0.072 | | | 1.24(1.01, 1.52), 0.043 | 1.21(0.98, 1.50), 0.078 | |
| ESV | 1.18(0.96, 1.46), 0.122 | | | 1.22(0.99, 1.53), 0.067 | 1.21(0.97, 1.52), 0.094 | |
| SV | 1.13(0.93, 1.38), 0.211 | | | 1.15(0.94, 1.39), 0.173 | 1.12(0.92, 1.36), 0.273 | |
| LVRI | 0.91(0.73, 1.12), 0.404 | | | 0.90(0.73, 1.1), 0.379 | 0.90(0.72, 1.1), 0.338 | |
| LVSI | 1.04(0.85, 1.28), 0.693 | | | 1.0(0.81, 1.24), 0.994 | 1.02(0.82, 1.26), 0.882 | |
| LV mass | 1.12(0.91, 1.38), 0.283 | | | 1.16(0.93, 1.45), 0.183 | 1.13(0.90, 1.41), 0.309 | |
| N=773 (97 composite cardiovascular endpoints, and 85 deaths of all-cause) for EF and volumes. N=747 (91 composite cardiovascular endpoints, and 82 deaths of all-cause) for LVRI, LVSI and LV mass. EF, SD=6.4%; EDV, SD=9.1ml/m^2^; ESV, SD=5.2ml/m^2^; SV, SD=5.7ml/m^2^; LVRI, SD=0.36g/ml; LVSI, SD=0.085; and LV mass, SD=12.7g/m^2^. Remaining footnotes and abbreviations are as in table-S3. | | | | | | |

| **Table-S6 Associations between 3DE LV measures and outcomes in men (complete case analysis)** | | | | |
| --- | --- | --- | --- | --- |
|  | **Unadjusted** | **Model-1** | **Model-2** | **Model-3** |
|  | **Standardized HR (95% CI), p value** | **Standardized HR (95% CI), p value** | **Standardized HR (95% CI), p value** | **Standardized HR (95% CI), p value** |
| **All-cause mortality** | | | | |
| EF | 0.78(0.66, 0.93), 0.005 | 0.78(0.65, 0.93), 0.005 | 0.78(0.65, 0.93), 0.005 | 0.80(0.67, 0.96), 0.020 |
| EDV | 1.23(1.04, 1.46), 0.017 | 1.26(1.06, 1.49), 0.008 | 1.26(1.06, 1.49), 0.009 | 1.22(1.03, 1.45), 0.022 |
| ESV | 1.34(1.11, 1.61), 0.002 | 1.37(1.14. 1.66), 0.001 | 1.37(1.14, 1.65), 0.001 | 1.31(1.09, 1.59), 0.005 |
| LVRI | 0.83(0.67, 1.02), 0.072 | 0.79(0.65, 0.97), 0.026 | 0.78(0.64, 0.95), 0.015 | 0.80(0.65, 0.98), 0.029 |
| LVSI | 1.30(1.09, 1.55), 0.003 | 1.28(1.06, 1.55), 0.011 | 1.27(1.05, 1.54), 0.015 | 1.28(1.05, 1.55), 0.011 |
| **Composite cardiovascular endpoint** | | |  |  |
| EF | 0.87(0.74, 1.02), 0.091 | 0.87(0.74, 1.02), 0.082 | 0.88(0.75, 1.02), 0.097 | 0.95(0.81, 1.11), 0.557 |
| EDV | 1.27(1.09, 1.48), 0.002 | 1.28(1.10, 1.49), 0.002 | 1.24(1.06, 1.44), 0.007 | 1.16(1.0, 1.35), 0.050 |
| ESV | 1.28(1.08, 1.51), 0.004 | 1.29(1.09, 1.52), 0.003 | 1.26(1.06, 1.48), 0.007 | 1.14(0.97, 1.35), 0.103 |
| LVRI | 0.82(0.68, 0.99), 0.035 | 0.85(0.70, 1.02), 0.086 | 0.85(0.70, 1.03), 0.096 | 0.89(0.74, 1.08), 0.254 |
| LVSI | 1.23(1.04, 1.44), 0.014 | 1.19(1.01, 1.41), 0.042 | 1.18(1.00, 1.39), 0.056 | 1.16(0.98, 1.37), 0.078 |
| N=707 (128 composite cardiovascular endpoints and 103 death of all-cause) for EF, EDV, and ESV. N=689 (121 composite cardiovascular endpoints and 99 deaths of all-cause) for LVRI and LVSI. EF, SD=7.0%; EDV, SD=10.0ml/m^2^; ESV, SD=6.1ml/m^2^; LVRI, SD=0.37g/ml; and LVSI, SD=0.086. Remaining footnotes and abbreviations are as in Table-S3. | | | | |

| **Table-S7 Associations between 3DE LV measures and outcomes in women (complete case analysis)** | | | | |
| --- | --- | --- | --- | --- |
|  | **Unadjusted** | **Model-1** | **Model-2** | **Model-3** |
|  | **Standardized HR (95% CI), p value** | **Standardized HR (95% CI), p value** | **Standardized HR (95% CI), p value** | **Standardized HR (95% CI), p value** |
| **All-cause mortality** | | | | |
| EF | 1.35(0.75, 2.43), 0.313 | 1.25(0.71, 2.19), 0.442 | 1.29(0.70, 2.36), 0.418 | 1.28(0.69, 2.37), 0.433 |
| EDV | 0.54(0.29, 1.01), 0.056 | 0.66(0.34, 1.28), 0.221 | 0.51(0.25, 1.07), 0.075 | 0.57(0.27, 1.18), 0.131 |
| ESV | 0.54(0.32, 0.93), 0.026 | 0.65(0.37, 1.14), 0.132 | 0.57(0.32, 1.02), 0.059 | 0.61(0.34, 1.08), 0.094 |
| LVRI | 1.57(1.0, 2.47), 0.052 | 1.37(0.86, 2.19), 0.188 | 1.69(1.02, 2.48), 0.042 | 1.53(0.91, 2.58), 0.112 |
| LVSI | 0.68(0.38, 1.21), 0.193 | 0.72(0.40, 1.28), 0.262 | 0.62(0.32, 1.18), 0.146 | 0.70(0.37, 1.33), 0.274 |
| **Composite cardiovascular endpoint** | |  |  |  |
| EF | 1.77(1.04, 3.0), 0.036 | 1.53(0.91, 2.58), 0.109 | 1.81(1.03, 3.17), 0.039 | 1.81(1.03, 3.17), 0.039 |
| EDV | 0.87(0.50, 1.51), 0.629 | 1.12(0.63, 2.0), 0.687 | 0.98(0.53, 1.81), 0.941 | 0.97(0.53, 1.81), 0.941 |
| ESV | 0.64(0.40, 1.03), 0.068 | 0.82(0.50, 1.33), 0.419 | 0.68(0.41, 1.13), 0.140 | 0.68(0.41, 1.13), 0.141 |
| LVRI | 1.31(0.85, 2.02), 0.216 | 1.16(0.77, 1.75), 0.487 | 1.22(0.81, 1.86), 0.345 | 1.22(0.80, 1.87), 0.340 |
| LVSI) | 0.73(0.45, 1.21), 0.225 | 0.73(0.45, 1.19), 0.206 | 0.66(0.39, 1.11), 0.118 | 0.65(0.38, 1.11), 0.116 |
| N=203 (22 composite cardiovascular endpoints, and 17 deaths of all-cause) for EF, EDV, and ESV. N=190 (20 composite cardiovascular endpoints and 16 deaths of all-cause) for LVRI and LVSI. EF, SD=5.6%; EDV, SD=7.3ml/m^2^; ESV, SD=3.7ml/m^2^; LVRI, SD=0.34g/ml; and LVSI, SD=0.087. Remaining footnotes and abbreviations are as in Table-S3. | | | | |

| Table-S8 Associations between 3DE LV measures and outcomes in men without CHD (complete case analysis) | | | | | | |
| --- | --- | --- | --- | --- | --- | --- |
|  | **Standardized HR (95% CI), p value** | | | | | |
|  | **Unadjusted** | | | **Model-1** | **Model-2** | |
| **All-cause mortality** | | | | |  | |
| EF | | 0.85(0.68, 1.1), 0.161 | 0.84(0.66, 1.1), 0.148 | | | 0.83(0.65, 1.1), 0.138 |
| EDV | | 1.20(0.97, 1.50), 0.116 | 1.24(1.0, 1.55), 0.056 | | | 1.27(1.01, 1.60), 0.036 |
| ESV | | 1.24(0.98, 1.58), 0.074 | 1.30(1.02. 1.66), 0.037 | | | 1.32(1.04, 1.69), 0.025 |
| LVRI | | 0.87(0.68, 1.11), 0.282 | 0.81(0.64, 1.0), 0.093 | | | 0.79(0.63, 1.0), 0.051 |
| LVSI | | 1.30(1.05, 1.60), 0.017 | 1.24(0.98, 1.56), 0.069 | | | 1.24(0.98, 1.56), 0.077 |
| **Composite cardiovascular endpoint** | | | | |  | |
| EF | 0.85(0.68, 1.06), 0.156 | | | 0.83(0.66, 1.03), 0.095 | 0.83(0.67, 1.03), 0.101 | |
| EDV | 1.22(0.98, 1.50), 0.072 | | | 1.26(1.01, 1.56), 0.037 | 1.24(1.0, 1.56), 0.060 | |
| ESV | 1.28(1.01, 1.61), 0.038 | | | 1.34(1.06, 1.69), 0.015 | 1.33(1.04, 1.69), 0.021 | |
| LVRI | 0.86(0.68, 1.1), 0.242 | | | 0.88(0.70, 1.1), 0.294 | 0.86(0.68, 1.1), 0.251 | |
| LVSI | 1.1(0.88, 1.37), 0.373 | | | 1.1(0.85, 1.33), 0.580 | 1.1(0.86, 1.37), 0.482 | |
| N=584 (79 composite cardiovascular endpoints, and 72 death of all-cause) for EF, EDV, and ESV. N=571 (75 composite cardiovascular endpoints, and 70 deaths of all-cause) for LVRI and LVSI. EF, SD=6.5%; EDV, SD=9.5ml/m2; ESV, SD=5.4ml/m2; LVRI, SD=0.38g/ml; and LVSI, SD=0.086. Remaining footnotes and abbreviations are as in Table-S3. | | | | | | |

| Table-S9 Associations between 3DE LV measures and outcomes in women without CHD (complete case analysis) | | | | | | |
| --- | --- | --- | --- | --- | --- | --- |
|  | **Standardized HR (95% CI), p value** | | | | | |
|  | **Unadjusted** | | | **Model-1** | **Model-2** | |
| **All-cause mortality** | | | | |  | |
| EF | | 1.46(0.75, 2.86), 0.266 | 1.33(0.70, 2.53), 0.378 | | | 1.25(0.62, 2.52), 0.530 |
| EDV | | 0.50(0.22, 1.1), 0.090 | 0.58(0.25, 1.34), 0.203 | | | 0.40(0.15, 1.06), 0.066 |
| ESV | | 0.50(0.25, 0.96), 0.038 | 0.57(0.29, 1.14), 0.116 | | | 0.55(0.27, 1.13), 0.103 |
| LVRI | | 1.28(0.67, 2.47), 0.452 | 1.15(0.59, 2.24), 0.679 | | | 1.72(0.83, 3.60), 0.145 |
| LVSI | | 0.73(0.37, 1.43), 0.357 | 0.71(0.35, 1.46), 0.355 | | | 0.67(0.30, 1.52), 0.341 |
| **Composite cardiovascular endpoint** | | | | |  | |
| EF | 1.85(1.03, 3.33), 0.041 | | | 1.55(0.87, 2.75), 0.133 | 1.68(0.90, 3.15), 0.107 | |
| EDV | 0.87(0.45, 1.66), 0.665 | | | 1.13(0.56, 2.3), 0.727 | 1.07(0.49, 2.33), 0.863 | |
| ESV | 0.61(0.34, 1.06), 0.083 | | | 0.77(0.43, 1.39), 0.396 | 0.73(0.40, 1.34), 0.309 | |
| LVRI | 1.15(0.65, 2.04), 0.629 | | | 1.1(0.62, 1.96), 0.764 | 1.1(0.63, 1.98), 0.711 | |
| LVSI | 0.78(0.45, 1.37), 0.401 | | | 0.70(0.37, 1.29), 0.246 | 0.68(0.36, 1.30), 0.245 | |
| N=189 (18 composite cardiovascular endpoints, and 13 deaths of all-cause) for EF, EDV, and ESV. N=176 (16 composite cardiovascular endpoints, and 12 deaths of all-cause) for LVRI and LVSI. EF, SD=5.7%; EDV, SD=6.9ml/m^2^; ESV, SD=3.6ml/m^2^; LVRI, SD=0.31g/ml; and LVSI, SD=0.083. Remaining footnotes and abbreviations are as in Table-S3. | | | | | | |

| Table-S10 Associations between 3DE LV measures and all-cause mortality and composite cardiovascular endpoint; role of diastolic function, macro-, and micro-vascular disease (imputed data) | | | | | |
| --- | --- | --- | --- | --- | --- |
|  | **All-cause mortality** | |  | **Composite cardiovascular endpoint** | |
|  | **Standardized HR (95% CI), p value** | | | | |
|  | **Men** | **Women** |  | **Men** | **Women** |
| **EF** |  |  |  | (SD=6.9%) | (SD=5.6%) |
| *Model-2* |  |  |  | 0.87(0.75, 1.02), 0.091 | 1.73(1.00, 3.00), 0.051 |
| ***Markers of diastolic dysfunction*** | |  |  |  |  |
| +3D Ed |  |  |  | 0.87(0.75, 1.02), 0.096 | 1.79(1.01, 3.19), 0.047 |
| +E/E’ |  |  |  | 0.87(0.75, 1.02), 0.085 | 2.06(1.14, 3.70), 0.016 |
| ***Markers of macro-vascular disease*** | |  |  |  |  |
| +3D TAC |  |  |  | 0.85(0.72, 1.0), 0.050 | 1.83(1.04, 3.23), 0.038 |
| *BSAi* |  |  |  | 0.85(0.72, 1.0), 0.045 | 1.86(1.04, 3.31), 0.036 |
| +3D Ea |  |  |  | 0.84(0.71, 0.99), 0.035 | 1.71(0.97, 3.00), 0.063 |
| *BSAi* |  |  |  | 0.83(0.70, 0.97), 0.022 | 1.71(0.97, 3.03), 0.064 |
| +Central AI |  |  |  | 0.88(0.75, 1.03), 0.108 | 1.77(1.00, 3.13), 0.049 |
| +PWV |  |  |  | 0.88(0.75, 1.03), 0.108 | 1.81(1.04, 3.15), 0.034 |
| ***Markers of micro-vascular disease*** | | | | | |
| +eGFR |  |  |  | 0.88(0.75, 1.03), 0.110 | 1.69(0.97, 2.93), 0.064 |
| +Microalbuminuria |  |  |  | 0.88(0.75, 1.02), 0.093 | 1.72(0.98, 3.01), 0.058 |
| +retinopathy |  |  |  | 0.86(0.74, 1.00), 0.058 | 1.66(0.95, 2.90), 0.074 |
| **EDV** | (SD=9.9ml/m^2^) | (SD=7.3ml/m^2^) |  | (SD=9.9ml/m^2^) | (SD=7.3ml/m^2^) |
| *Model-2* | 1.25(1.05, 1.48), 0.010 | 0.54(0.26, 1.10), 0.092 |  | 1.24(1.06, 1.45), 0.006 | 1.03(0.56, 1.91), 0.913 |
| ***Markers of diastolic dysfunction*** | | | | | |
| +E/E’ | 1.24(1.05, 148), 0.013 | 0.38(0.17, 0.84), 0.018 |  | 1.24(1.06, 1.44), 0.008 | 0.83(0.44, 1.57), 0.569 |
| ***Markers of macro-vascular disease*** | |  |  |  |  |
| +TAC *BSAi* | 1.24(0.99, 1.55), 0.065 | 0.71(0.28, 1.82), 0.480 |  | 1.33(1.10, 1.62), 0.004 | 1.10(0.50, 2.39), 0.822 |
| +Ea *BSAi* | 1.45(1.15,1.84), 0.002 | 0.61(0.13, 2.73), 0.513 |  | 1.33(1.08, 1.64), 0.007 | 0.56(0.15, 2.04), 0.380 |
| +Central AI | 1.25(1.06, 1.49), 0.010 | 0.54(0.26, 1.11), 0.093 |  | 1.21(1.04, 1.42), 0.016 | 1.03(0.56, 1.92), 0.908 |
| +PWV | 1.25(1.05, 1.48), 0.011 | 0.54(0.26, 1.11), 0.092 |  | 1.24(1.07, 1.45), 0.005 | 1.03(0.55, 1.94), 0.908 |
| **ESV** | (SD=6.1ml/m^2^) | (SD=3.7ml/m^2^) |  | (SD=6.1ml/m^2^) | (SD=3.7ml/m^2^) |
| *Model-2* | 1.36(1.12, 1.63), 0.001 | 0.59(0.33, 1.04), 0.070 |  | 1.26(1.07, 1.49), 0.006 | 0.72(0.43, 1.18), 0.199 |
| ***Markers of diastolic dysfunction*** | |  |  |  |  |
| +E/E’ | 1.35(1.12, 1.63), 0.002 | 0.46(0.25, 0.86), 0.015 |  | 1.26(1.06, 1.49), 0.007 | 0.59(0.35, 0.99), 0.044 |
| ***Markers of macro-vascular disease*** | | |  |  |  |
| +TAC *BSAi* | 1.32(1.09, 1.61), 0.005 | 0.66(0.36, 1.21), 0.178 |  | 1.26(1.06, 1.50), 0.009 | 0.71(0.42, 1.20), 0.197 |
| +Ea *BSAi* | 1.38(1.13, 1.69), 0.001 | 0.67(0.35, 1,28), 0.222 |  | 1.25(1.04, 1.50), 0.014 | 0.59(0.33, 1.05), 0.072 |
| +Central AI | 1.36(1.13, 1.64), 0.001 | 0.57(0.32, 1.03), 0.063 |  | 1.24(1.04, 1.46), 0.014 | 0.72(0.43, 1.19), 0.204 |
| +PWV | 1.35(1.12, 1.63), 0.002 | 0.58(0.32, 1.04), 0.069 |  | 1.26(1.06, 1.48), 0.007 | 0.69(0.41, 1.15), 0.157 |
| **LVRI** | (SD=0.37g/ml) | (SD=0.34g/ml) |  | (SD=0.37g/ml) | (SD=0.34g/ml) |
| *Model-2* | 0.79(0.65, 0.96), 0.019 | 1.70(1.03, 2.80), 0.038 |  | 0.85(0.70, 1.02), 0.086 | 1.25(0.83, 1.87), 0.280 |
| ***Markers of diastolic dysfunction*** | | | | | |
| +E/E’ | 0.79(0.64, 0.96), 0.019 | 1.68(0.99, 2.85), 0.055 |  | 0.85(0.70, 1.02), 0.087 | 1.38(0.90, 2.12), 0.135 |
| ***Markers of macro-vascular disease*** | |  |  |  |  |
| +TAC *BSAi* | 0.83(0.67, 1.04), 0.102 | 1.50(0.85, 2.65), 0.157 |  | 0.86(0.70, 1.05), 0.138 | 1.30(0.82, 2.08), 0.262 |
| +Ea *BSAi* | 0.80(0.63, 1.01), 0.060 | 1.55(0.80, 3.04), 0.194 |  | 0.89(0.71, 1.11), 0.315 | 1.65(0.93, 2.93), 0.084 |
| +Central AI | 0.79(0.64, 0.96), 0.019 | 1.71(1.03, 2.82), 0.038 |  | 0.85(0.70, 1.03), 0.096 | 1.25(0.83, 1.88), 0.283 |
| +PWV | 0.79(0.65, 0.97), 0.023 | 1.71(1.04, 2.83), 0.035 |  | 0.85(0.70, 1.03), 0.097 | 1.18(0.77, 1.80), 0.447 |
| **LVSI** | (SD=0.086) | (SD=0.087) |  | (SD=0.086) | (SD=0.087) |
| *Model-2* | 1.26(1.05, 1.54), 0.015 | 0.61(0.32, 1.15), 0.129 |  | 1.18(1.0, 1.39), 0.047 | 0.63(0.37, 1.06), 0.079 |
| ***Markers of diastolic dysfunction*** | | | | | |
| +E/E’ | 1.27(1.05, 1.54), 0.016 | 0.63(0.31, 1.28), 0.200 |  | 1.18(1.00, 1.40), 0.047 | 0.62(0.34, 1.15), 0.129 |
| ***Markers of macro-vascular disease*** | |  |  |  |  |
| +TAC *BSAi* | 1.22(0.99, 1.49), 0.057 | 0.70(0.37, 1.31), 0.258 |  | 1.17(0.99, 1.39), 0.070 | 0.60(0.34, 1.05), 0.074 |
| +Ea *BSAi* | 1.24(1.0, 1.52), 0.043 | 0.70(0.37, 1.33), 0.277 |  | 1.15(0.96, 1.37), 0.126 | 0.54(0.29, 0.99), 0.047 |
| +Central AI | 1.26(1.05, 1.54), 0.015 | 0.61(0.32, 1.16), 0.135 |  | 1.18(1.00, 1.39), 0.056 | 0.63(0.38, 1.06), 0.084 |
| +PWV | 1.27(1.05, 1.54), 0.016 | 0.61(0.33, 1.15), 0.125 |  | 1.19(1.00, 1.40), 0.042 | 0.62(0.35, 1.08), 0.089 |
| Men, N=717(105 deaths and 128 composite cardiovascular endpoints) for EDV and ESV; and N=699(101 deaths and 121 composite cardiovascular endpoints) for LVRI and LVSI. Women, N=205(18 deaths and 23 composite cardiovascular endpoints) for EDV and ESV; and N=192(17 deaths and 21 composite cardiovascular endpoints) for LVRI and LVSI. Model-2: adjusted for age, ethnicity, systolic blood pressure, antihypertensive medication, cholesterol: HDL ratio, body mass index, diabetes mellitus, and smoking. Abbreviations: AI, augmentation index; BSAi, body surface area indexed; Ea, effective arterial elastance; Ed, LV diastolic stiffness; EDV, end-diastolic volumes; ESV, end-systolic volumes; HR, hazard ratio; LV, left ventricular; EF, ejection fraction; LVRI, LV remodeling index; LVSI, LV sphericity index; SD, standard deviation; PWV, pulse wave velocity; and TAC, total arterial compliance. Data for EF and all-cause mortality are not shown for this analysis as there was no evidence for significant sex-interaction (see Table-2). | | | | | |

| **Table-S11 Associations between 3DE LV measures and all-cause mortality and composite cardiovascular endpoint; role of diastolic function (compete case analysis)** | | | | | |
| --- | --- | --- | --- | --- | --- |
|  | **All-cause mortality** | |  | **Composite cardiovascular endpoint** | |
|  | **Standardized HR (95% CI), p value** | | | | |
|  | **Men** | **Women** |  | **Men** | **Women** |
| **EF** |  |  |  | (SD=7.0%) | (SD=5.6%) |
| *Model-2* |  |  |  | 0.88(0.75, 1.03), 0.114 | 1.80(1.02, 3.15), 0.041 |
| +3D Ed |  |  |  | 0.88(0.75, 1.03), 0.120 | 1.82(1.02, 3.25), 0.044 |
| +E/E’ |  |  |  | 0.88(0.75, 1.03), 0.107 | 2.05(1.14, 3.68), 0.017 |
| **EDV** | (SD=9.9ml/m^2^) | (SD=7.3ml/m^2^) |  | (SD=9.9ml/m^2^) | (SD=7.3ml/m^2^) |
| *Model-2* | 1.26(1.07, 1.50), 0.007 | 0.52(0.25, 1.07), 0.075 |  | 1.22(1.05, 1.43), 0.011 | 0.98(0.53, 1.81), 0.038 |
| +3D Ed | 1.27(1.05, 1.54), 0.015 | 0.90(0.41, 1.94), 0.779 |  | 1.28(1.08, 1.53), 0.006 | 1.39(0.73, 2.64), 0.313 |
| +E/E’ | 1.26(1.06, 149), 0.009 | 0.32(0.14, 0.78), 0.011 |  | 1.22(1.04, 1.43), 0.014 | 0.83(0.44, 1.57), 0.571 |
| **ESV** | (SD=6.1ml/m^2^) | (SD=3.7ml/m^2^) |  | (SD=6.1ml/m^2^) | (SD=3.7ml/m^2^) |
| *Model-2* | 1.38(1.15, 1.67), 0.001 | 0.57(0.32, 1.03), 0.061 |  | 1.24(1.05, 1.47), 0.013 | 0.69(0.42, 1.14), 0.145 |
| +3D Ed | 1.41(1.14, 1.73), 0.001 | 0.83(0.45, 1.52), 0.540 |  | 1.29(1.07, 1.55), 0.008 | 0.85(0.50, 1.44), 0.541 |
| + E/E’ | 1.38(1.14, 1.66), 0.001 | 0.42(0.22, 0.81), 0.010 |  | 1.24(1.04, 1.47), 0.014 | 0.59(0.35, 0.99), 0.046 |
| **LVRI** | (SD=0.37g/ml) | (SD=0.34g/ml) |  | (SD=0.37g/ml) | (SD=0.34g/ml) |
| *Model-2* | 0.78(0.64, 0.95), 0.015 | 1.69(1.02, 2.80), 0.041 |  | 0.87(0.72, 1.05), 0.150 | 1.22(0.81, 1.86), 0.338 |
| +3D Ed | 0.83(0.67, 1.03), 0.085 | 1.10(0.65, 1.79), 0.760 |  | 0.90(0.73, 1.11), 0.314 | 0.96(0.62, 1.48), 0.850 |
| +E/E’ | 0.78(0.64, 0.95), 0.015 | 1.99(1.16, 3.39), 0.012 |  | 0.87(0.72, 1.05), 0.150 | 1.36(0.88, 2.09), 0.165 |
| **LVSI** | (SD=0.086) | (SD=0.087) |  | (SD=0.086) | (SD=0.087) |
| *Model-2* | 1.28(1.05, 1.55), 0.015 | 0.62(0.32, 1.18), 0.145 |  | 1.15(0.97, 1.37), 0.108 | 0.66(0.39, 1.11), 0.117 |
| +3D Ed | 1.21(1.0, 1.49), 0.062 | 0.77(0.38, 1.58), 0.477 |  | 1.12(0.94, 1.34), 0.195 | 0.80(0.45, 1.43), 0.459 |
| +E/E’ | 1.27(1.05, 1.55), 0.015 | 0.51(0.24, 1.10), 0.085 |  | 1.15(0.97, 1.37), 0.106 | 0.64(0.35, 1.16), 0.141 |
| Men, N=696 (102 deaths and 124 composite cardiovascular endpoints) for EDV and ESV; and N=679 (98 deaths and 117 composite cardiovascular endpoints) for LVRI and LVSI. Women, N=201 (17 deaths and 22 composite cardiovascular endpoints) for EDV and ESV; and N=188 (16 deaths and 20 composite cardiovascular endpoints) for LVRI and LVSI. Remaining footnotes and abbreviations are as in table-S10. Data for EF and all-cause mortality are not shown for this analysis as there was no evidence for significant sex-interaction (see table-2). | | | | | |

| **Table-S12 Associations between 3DE LV measures and all-cause mortality and composite cardiovascular endpoints; role of macro-vascular disease (complete case analysis)** | | | | | |
| --- | --- | --- | --- | --- | --- |
|  | **All-cause mortality** | |  | **Composite cardiovascular endpoint** | |
|  | **Standardized HR (95% CI), p value** | | | | |
|  | **Men** | **Women** |  | **Men** | **Women** |
| **EF** |  |  |  | (SD=7.0%) | (SD=5.6%) |
| *Model-2* |  |  |  | 0.88(0.75, 1.02), 0.097 | 1.81(1.03, 3.17), 0.039 |
| +3D TAC |  |  |  | 0.85(0.73, 1.00), 0.056 | 1.92(1.08, 3.42), 0.027 |
| *BSAi* |  |  |  | 0.85(0.72, 1.00), 0.050 | 1.95(1.08, 3.50), 0.026 |
| +3D Ea |  |  |  | 0.84(0.71, 0.99), 0.038 | 1.80(1.02, 3.19), 0.044 |
| *BSAi* |  |  |  | 0.83(0.70, 0.98), 0.024 | 1.82(1.01, 3.24), 0.043 |
| *Model-2* |  |  |  | 0.85(0.73, 1.00), 0.052 | 1.70(0.97, 2.99), 0.064 |
| +Central AI |  |  |  | 0.86(0.73, 1.01), 0.059 | 1.69(0.94, 3.02), 0.078 |
| *Model-2* |  |  |  | 0.86(0.71, 1.04), 0.119 | 2.33(1.22, 4.44), 0.011 |
| + PWV |  |  |  | 0.87(0.72, 1.05), 0.140 | 2.33(1.22, 4.47), 0.011 |
| **EDV** | (SD=9.9ml/m^2^) | (SD=7.3ml/m^2^) |  | (SD=9.9ml/m^2^) | (SD=7.3ml/m^2^) |
| *Model-2* | 1.26(1.06, 1.49), 0.009 | 0.51(0.25, 1.07), 0.075 |  | 1.24(1.06, 1.44), 0.007 | 0.98(0.53, 1.81), 0.941 |
| +3D TAC | 1.21(0.98, 1.50), 0.083 | 0.73(0.28, 1.95), 0.537 |  | 1.32(1.09, 1.59), 0.004 | 1.06(0.48, 2.34), 0.890 |
| *BSAi* | 1.23(0.98, 1.55), 0.069 | 0.69(0.26, 1.82), 0.449 |  | 1.33(1.09, 1.61), 0.004 | 1.01(0.46, 2.23), 0.981 |
| +3D Ea | 1.40(1.11, 1.77), 0.004 | 0.59(0.13, 2.63), 0.493 |  | 1.36(1.11, 1.65), 0.003 | 0.55(0.16, 1.92), 0.353 |
| *BSAi* | 1.47(1.16, 1.85), 0.001 | 0.56(0.12, 2.64), 0.464 |  | 1.32(1.08, 1.63), 0.008 | 0.53(0.14, 1.93), 0.334 |
| *Model-2* | 1.26(1.05, 1.50), 0.012 | 0.69(0.29, 1.66), 0.409 |  | 1.23(1.05, 1.44), 0.011 | 1.27(0.63, 2.57), 0.501 |
| +Central AI | 1.25(1.05, 1.50), 0.013 | 0.69(0.29, 1.67), 0.414 |  | 1.20(1.02, 1.41), 0.027 | 1.31(0.64, 2.66), 0.458 |
| *Model-2* | 1.16(0.92, 1.45), 0.201 | 0.43(0.17, 1.07), 0.068 |  | 1.30(1.07, 1.59), 0.010 | 1.06(0.52, 2.14), 0.878 |
| + PWV | 1.16(0.93, 1.45), 0.195 | 0.43(0.17, 1.08), 0.073 |  | 1.31(1.08, 1.60), 0.007 | 1.05(0.52, 2.11), 0.898 |
| **ESV** | (SD=6.1ml/m^2^) | (SD=3.7ml/m^2^) |  | (SD=6.1ml/m^2^) | (SD=3.7ml/m^2^) |
| *Model-2* | 1.37(1.14, 1.65), 0.001 | 0.57(0.32, 1.02), 0.059 |  | 1.26(1.06, 1.48), 0.007 | 0.68(0.41, 1.13), 0.140 |
| +3D TAC | 1.33(1.09, 1.62), 0.004 | 0.67(0.36, 1.26), 0.214 |  | 1.26(1.06, 1.49), 0.009 | 0.67(0.39, 1.15), 0.147 |
| *BSAi* | 1.34(1.09, 1.63), 0.004 | 0.65(0.35, 1.19), 0.165 |  | 1.26(1.06, 1.49), 0.010 | 0.67(0.39, 1.14), 0.138 |
| +3D Ea | 1.39(1.14, 1.70), 0.001 | 0.66(0.34, 1.29), 0.223 |  | 1.26(1.05, 1.50), 0.011 | 0.54(0.30, 0.99), 0.047 |
| *BSAi* | 1.40(1.15, 1.71), 0.001 | 0.65(0.34, 1.26), 0.205 |  | 1.24(1.04, 1.48), 0.016 | 0.55(0.31, 1.01), 0.052 |
| *Model-2* | 1.38(1.14, 1.68), 0.001 | 0.73(0.38, 1.41), 0.353 |  | 1.27(1.07, 1.50), 0.007 | 0.80(0.46, 1.39), 0.432 |
| +Central AI | 1.38(1.14, 1.68), 0.001 | 0.73(0.37, 1.42), 0.349 |  | 1.24(1.04, 1.48), 0.015 | 0.82(0.47, 1.45), 0.500 |
| *Model-2* | 1.27(1.00, 1.61), 0.055 | 0.42(0.20, 0.89), 0.023 |  | 1.32(1.07, 1.64), 0.011 | 0.63(0.35, 1.13), 0.123 |
| +PWV | 1.27(1.00, 1.61), 0.054 | 0.44(0.21, 0.94), 0.034 |  | 1.32(1.06, 1.63), 0.022 | 0.64(0.35, 1.15), 0.136 |
| **LVRI** | (SD=0.37g/ml) | (SD=0.34g/ml) |  | (SD=0.37g/ml) | (SD=0.34g/ml) |
| *Model-2* | 0.78(0.64, 0.95), 0.015 | 1.69(1.02, 2.80), 0.042 |  | 0.85(0.70, 1.03), 0.096 | 1.22(0.81, 1.86), 0.345 |
| +3D TAC | 0.83(0.67, 1.03), 0.098 | 1.42(0.78, 2.59), 0.246 |  | 0.86(0.70, 1.05), 0.132 | 1.24(0.76, 2.02), 0.390 |
| *BSAi* | 0.83(0.66, 1.03), 0.094 | 1.48(0.83, 2.63), 0.184 |  | 0.86(0.70, 1.05), 0.146 | 1.27(0.79, 2.05), 0.322 |
| +3D Ea | 0.80(0.63, 1.01), 0.063 | 1.51(0.73, 3.16), 0.267 |  | 0.88(0.71, 1.09), 0.243 | 1.57(0.87, 2.83), 0.137 |
| *BSAi* | 0.78(0.62, 1.00), 0.046 | 1.50(0.75, 3.01), 0.248 |  | 0.89(0.72, 1.11), 0.324 | 1.52(0.85, 2.72), 0.159 |
| *Model-2* | 0.82(0.67, 2.02), 0.067 | 1.34(0.72, 2.52), 0.356 |  | 0.87(0.71, 1.06), 0.165 | 0.95(0.56, 1.62), 0.863 |
| +Central AI | 0.82(0.67, 1.02), 0.070 | 1.34(0.71, 2.52), 0.366 |  | 0.87(0.72, 1.06), 0.177 | 0.94(0.55, 1.59), 0.808 |
| *Model-2* | 0.92(0.72, 1.17), 0.509 | 1.66(0.90, 3.06), 0.104 |  | 0.83(0.65, 1.05), 0.118 | 1.10(0.68, 1.78), 0.690 |
| +PWV | 0.93(0.73, 1.18), 0.545 | 1.79(0.96, 3.35), 0.068 |  | 0.83(0.66, 1.05), 0.123 | 1.11(0.68, 1.82), 0.675 |
| **LVSI** | (SD=0.086) | (SD=0.087) |  | (SD=0.086) | (SD=0.087) |
| *Model-2* | 1.27(1.05, 1.54), 0.015 | 0.62(0.32, 1.18), 0.146 |  | 1.18(1.00, 1.39), 0.056 | 0.66(0.39, 1.11), 0.118 |
| +3D TAC | 1.22(0.99, 1.49), 0.058 | 0.75(0.39, 1.44), 0.386 |  | 1.17(0.98, 1.39), 0.074 | 0.63(0.36, 1.13), 0.125 |
| *BSAi* | 1.22(0.99, 1.49), 0.058 | 0.71(0.37, 1.36), 0.301 |  | 1.17(0.98, 1.38), 0.080 | 0.63(0.36, 1.11), 0.109 |
| +3D Ea | 1.23(1.01, 1.51), 0.044 | 0.73(0.38, 1.42), 0.360 |  | 1.15(0.97, 1.37), 0.113 | 0.56(0.30, 1.05), 0.073 |
| *BSAi* | 1.24(1.01, 1.52), 0.040 | 0.73(0.38, 1.40), 0.341 |  | 1.14(0.96, 1.36), 0.140 | 0.58(0.32, 1.07), 0.083 |
| *Model-2* | 1.21(0.97, 1.50), 0.083 | 0.59(0.25, 1.39), 0.225 |  | 1.16(0.97, 1.38), 0.097 | 0.83(0.45, 1.53), 0.548 |
| +Central AI | 1.21(0.97, 1.50), 0.087 | 0.59(0.25, 1.43), 0.245 |  | 1.15(0.96, 1.38), 0.121 | 0.85(0.46, 1.58), 0.613 |
| *Model-2* | 1.19(0.94, 1.52), 0.153 | 0.43(0.19, 0.99), 0.046 |  | 1.21(0.99, 1.49), 0.067 | 0.73(0.41, 1.27), 0.263 |
| +PWV | 1.19(0.94, 1.52), 0.152 | 0.48(0.22, 1.06), 0.070 |  | 1.23(1.00, 1.50), 0.052 | 0.72(0.41, 1.28), 0.265 |
| Men, N=707(103 deaths and 128 composite cardiovascular endpoints) for EDV and ESV; and N=689(99 deaths and 121 composite cardiovascular endpoints) for LVRI and LVSI. Women, N=203(17 deaths and 22 for composite cardiovascular endpoints) for EDV and ESV; and N=190(16 deaths and 20 composite cardiovascular endpoints) for LVRI and LVSI.  For models with central AI: men, N=644(92 deaths and 117 composite cardiovascular endpoints) and women, N=183(14 deaths and 20 composite cardiovascular endpoints) for EDV and ESV; men, N=628(89 deaths and 111 composite cardiovascular endpoints) and women, N=170(13 deaths and 18 composite cardiovascular endpoints) for LVRI and LVSI.  For models with PWV: men, N=536(71 deaths and 89 composite cardiovascular endpoints) and women, N=156(10 deaths and 17 composite cardiovascular endpoints) for EDV and ESV; men, N=527(70 deaths and 85 composite cardiovascular endpoints) and women, N=145(9 deaths and 15 composite cardiovascular endpoints) for LVRI and LVSI. Remaining footnotes and aabbreviations are as in table-S10. Data for EF and all-cause mortality are not shown for this analysis as there was no evidence for significant sex-interaction (see table-2). | | | | | |

| **Table-S13 Associations between 3DE LV measures and all-cause mortality and composite cardiovascular endpoint; role of micro-vascular disease (complete case analysis)** | | | | | |
| --- | --- | --- | --- | --- | --- |
|  | **All-cause mortality** | |  | **Composite cardiovascular endpoint** | |
|  | **Standardized HR (95% CI), p value** | | | | |
|  | **Men** | **Women** |  | **Men** | **Women** |
| **EF** |  |  |  | (SD=7.0%) | (SD=5.6%) |
| *Model 2* |  |  |  | 0.88(0.76, 1.03), 0.116 | 1.81(1.03, 3.17), 0.038 |
| +eGFR |  |  |  | 0.89(0.76, 1.04), 0.135 | 1.80(1.02, 3.18), 0.044 |
| +Microalbuminuria |  |  |  | 0.88(0.76, 1.03), 0.119 | 1.80(1.02, 3.19), 0.043 |
| *Model 2* |  |  |  | 0.93(0.79, 1.11), 0.446 | 2.08(1.06, 4.07), 0.033 |
| +retinopathy |  |  |  | 0.92(0.77, 1.09), 0.346 | 1.96(1.0, 3.84), 0.049 |
| **EDV** | (SD=9.9ml/m^2^) | (SD=7.3ml/m^2^) |  | (SD=9.9ml/m^2^) | (SD=7.3ml/m^2^) |
| *Model 2* | 1.25(1.05, 1.48), 0.010 | 0.51(0.25, 1.07), 0.075 |  | 1.23(1.05, 1.44), 0.009 | 0.98(0.53, 1.81), 0.943 |
| +eGFR | 1.23(1.04, 1.45), 0.015 | 0.51(0.25, 1.05), 0.067 |  | 1.23(1.05, 1.43), 0.009 | 0.98(0.53, 1.80), 0.937 |
| +Microalbuminuria | 1.24(1.05, 1.48), 0.012 | 0.59(0.27, 1.28), 0.179 |  | 1.23(1.05, 1.44), 0.008 | 1.15(0.60, 2.21), 0.665 |
| *Model 2* | 1.30(1.07, 1.58), 0.007 | 0.34(0.14, 0.81), 0.015 |  | 1.25(1.04, 1.49), 0.016 | 1.03(0.49, 2.13), 0.943 |
| +retinopathy | 1.30(1.07, 2.58), 0.007 | 0.35(0.14, 0.82), 0.016 |  | 1.26(1.05, 1.50), 0.012 | 0.96(0.45, 2.04), 0.913 |
| **ESV** | (SD=6.1ml/m^2^) | (SD=3.7ml/m^2^) |  | (SD=6.1ml/m^2^) | (SD=3.7ml/m^2^) |
| *Model 2* | 1.36(1.13, 1.64), 0.001 | 0.57(0.32, 1.02), 0.059 |  | 1.25(1.05, 1.48), 0.010 | 0.68(0.41, 1.13), 0.140 |
| +eGFR | 1.33(1.11, 1.60), 0.002 | 0.58(0.33, 1.03), 0.064 |  | 1.24(1.05, 1.47), 0.010 | 0.69(0.42, 1.14), 0.151 |
| +Microalbuminuria | 1.36(1.13, 1.64), 0.001 | 0.60(0.33, 1.11), 0.103 |  | 1.25(1.06, 1.47), 0.009 | 0.74(0.44, 1.25), 0.264 |
| *Model 2* | 1.38(1.12, 1,71), 0.003 | 0.42(0.20, 0.90), 0.025 |  | 1.22(1.01, 1.48), 0.039 | 0.63(0.34, 1.16), 0.135 |
| +retinopathy | 1.38(1.12, 1.71), 0.003 | 0.42(0.19, 0.90), 0.025 |  | 1.24(1.03, 1.50), 0.025 | 0.62(0.33, 1.14), 0.125 |
| **LVRI** | (SD=0.37g/ml) | (SD=0.34g/ml) |  | (SD=0.37g/ml) | (SD=0.34g/ml) |
| *Model 2* | 0.78(0.64, 0.96), 0.017 | 1.69(1.02, 2.80), 0.042 |  | 0.85(0.71, 1.03), 0.106 | 1.22(0.81, 1.86), 0.346 |
| +eGFR | 0.79(0.65, 0.97), 0.021 | 1.74(1.04, 2.91), 0.035 |  | 0.86(0.71, 1.04), 0.114 | 1.22(0.80, 1.86), 0.351 |
| +Microalbuminuria | 0.79(0.64, 0.96), 0.020 | 1.77(1.04, 3.00), 0.035 |  | 0.85(0.70, 1.03), 0.095 | 1.21(0.78, 1.87), 0.397 |
| *Model 2* | 0.74(0.59, 0.93), 0.010 | 2.08(1.06, 4.10), 0.034 |  | 0.86(0.69, 1.06), 0.153 | 1.26(0.78, 2.04), 0.338 |
| +retinopathy | 0.74(0.59, 0.93), 0.011 | 2.06(1.01, 4.19), 0.046 |  | 0.86(0.69, 1.06), 0.158 | 1.48(0.88, 2.50), 0.141 |
| **LVSI** | (SD=0.086) | (SD=0.087) |  | (SD=0.086) | (SD=0.087) |
| *Model 2* | 1.27(1.04, 1.54), 0.017 | 0.62(0.32, 1.18), 0.146 |  | 1.17(0.99, 1.38), 0.067 | 0.66(0.39, 1.11), 0.119 |
| +eGFR | 1.29(1.06, 1.56), 0.011 | 0.63(0.33, 1.21), 0.167 |  | 1.17(0.99, 1.38), 0.059 | 0.66(0.39, 1.12), 0.122 |
| +Microalbuminuria | 1.27(1.05, 1.54), 0.016 | 0.70(0.35, 1.38), 0.303 |  | 1.17(0.99, 1.38), 0.066 | 0.74(0.43, 1.26), 0.265 |
| *Model 2* | 1.31(1.04, 1.64), 0.019 | 0.71(0.29, 1.72), 0.445 |  | 1.16(0.96, 1.41), 0.118 | 0.56(0.28, 1.14), 0.112 |
| +retinopathy | 1.31(1.04, 1.64), 0.021 | 0.71(0.29, 1.76), 0.463 |  | 1.16(0.96, 1.41), 0.121 | 0.54(0.27, 1.11), 0.093 |

Men, N=702(103 deaths and 128 composite cardiovascular endpoints) for EDV and ESV, and N=689(99 deaths and 121 composite cardiovascular endpoints) for LVRI and LVSI. Women, N=201(17 deaths and 22 composite cardiovascular endpoints) for EDV and ESV, and N=188(16 deaths and 20 composite cardiovascular endpoints) for LVRI and LVSI. For models with retinopathy: men, N=603(84 deaths and 106 composite cardiovascular endpoints) and women, N=158(12 deaths and 17 composite cardiovascular endpoints) for EDV and ESV; men, N=590(82 deaths and 103 composite cardiovascular endpoints) and women, N=147(11 deaths and 15 composite cardiovascular endpoints) for LVRI and LVSI. Remaining footnotes and abbreviations are as in table-S10 in addition to eGFR, estimated glomerular filtration rate.
