# Supplementary material for: Sex-differences in associations of LV structure and function measured by echocardiography with long-term risk of mortality and cardiovascular morbidity
Source: Front Cardiovasc Med. 2023 Apr 25;10:1144964. doi: 10.3389/fcvm.2023.1144964 (PMC10166834; doi:10.3389/fcvm.2023.1144964)
Supplement: Supplementary file 2 [file Table2.docx]

**
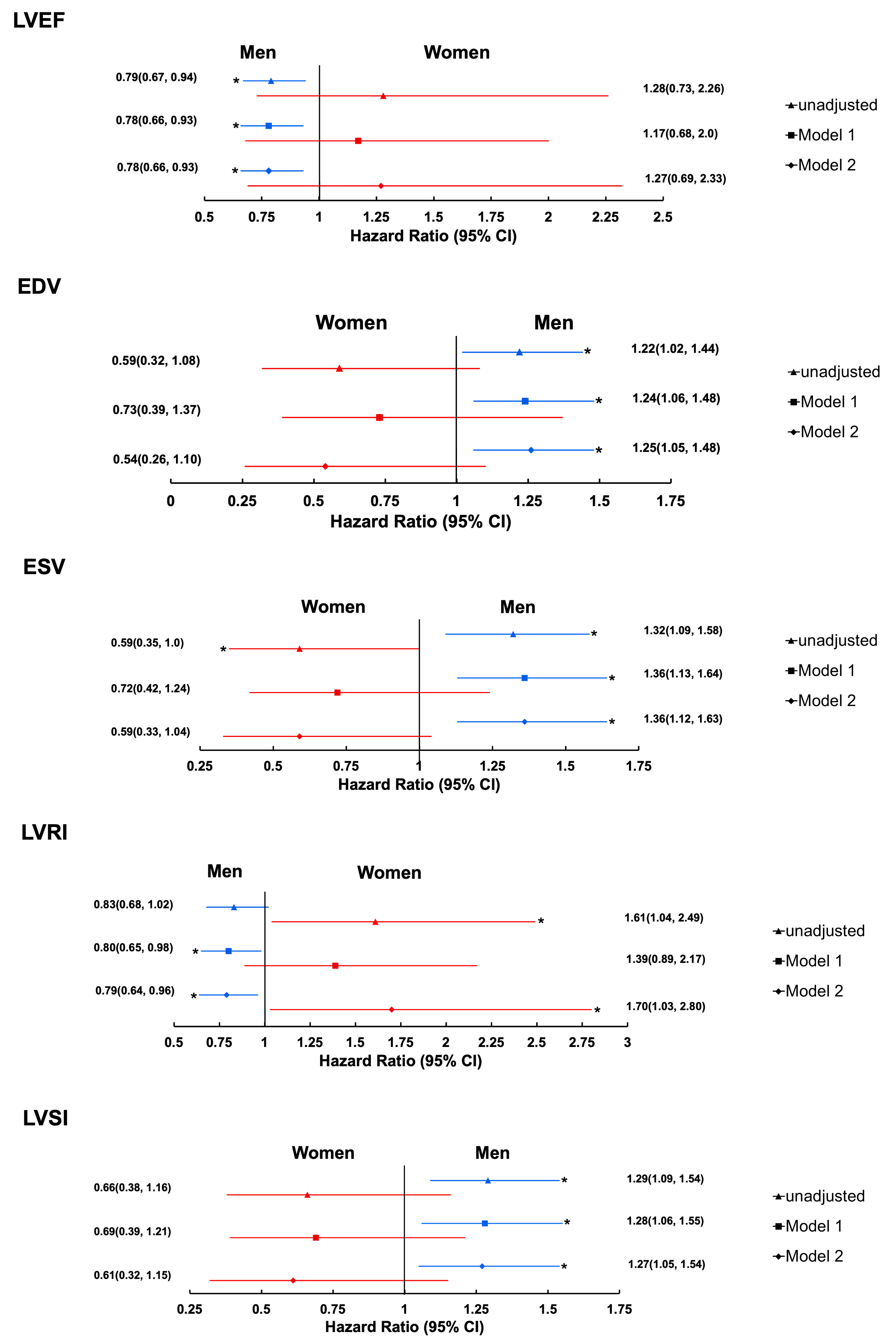
**

**A**


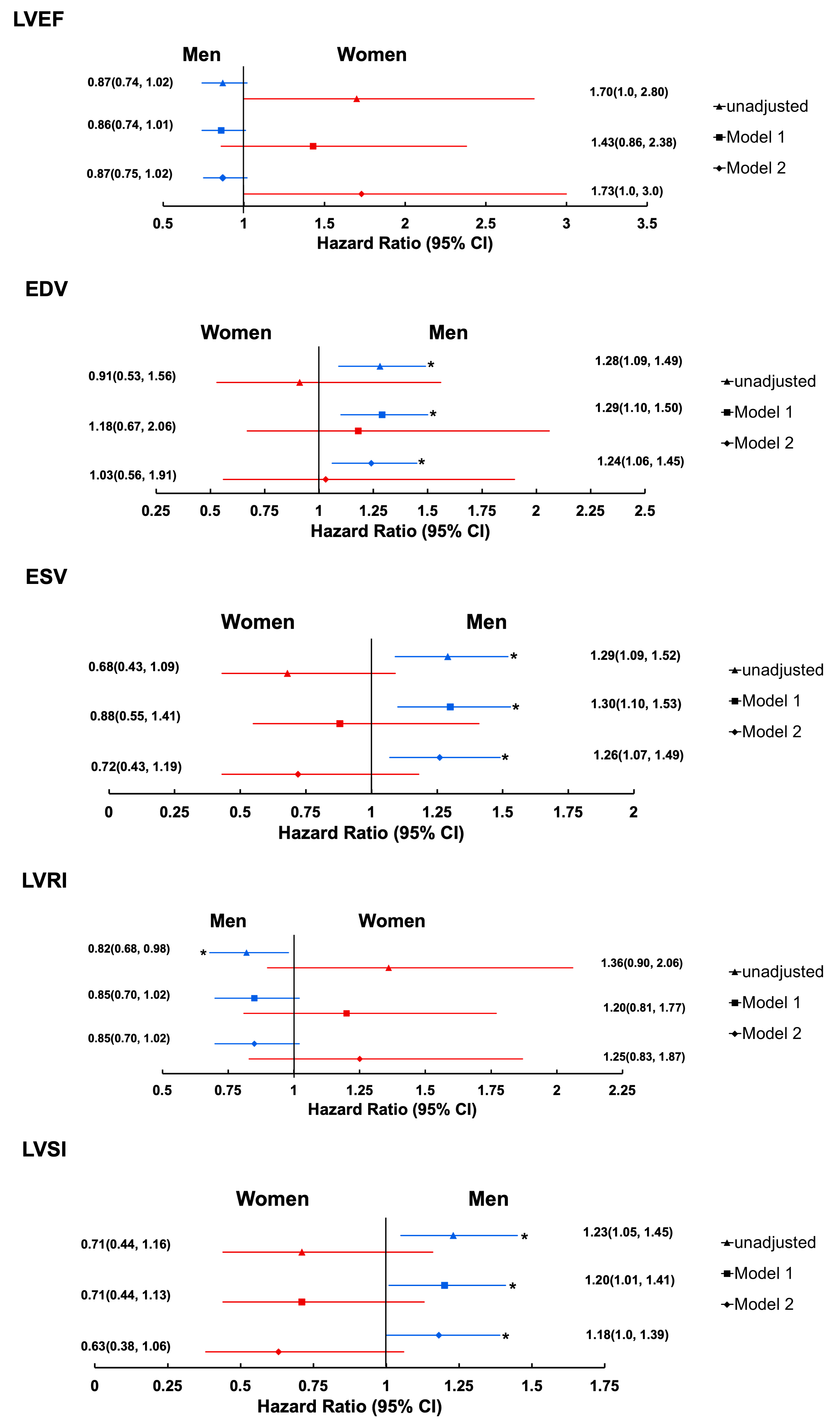


**B**

**Figure-1 Forest plots showing the associations between 3DE LV measures, and all-cause mortality(A) and composite cardiovascular endpoint(B) stratified by sex(imputed data).** Estimates are HR(95% CI) per SD change. Model-1: adjusted for age and ethnicity. Model-2: model-1 + systolic blood pressure, antihypertensive medication, cholesterol: HDL ratio, body mass index, diabetes mellitus, and smoking. 3D ESV is log transformed. Men: N=717(128 composite cardiovascular endpoints and 105 deaths of all-cause) for EF, EDV, and ESV; N=699(121 composite cardiovascular endpoints and 101 death of all-causes) for LVRI and LVSI (EF, SD=6.9%; EDV, SD=9.9ml/m^2^; ESV, SD=6.1ml/m^2^; LVRI, SD=0.37g/ml; and LVSI, SD=0.086). Women: N=205(23 composite cardiovascular endpoints and 18 deaths of all-cause) for EF, EDV, and ESV; N=192(21 composite cardiovascular endpoints and 17 deaths of all-cause) for LVRI and LVSI (EF, SD=5.6%; EDV, SD=7.3ml/m^2^; ESV, SD=3.7ml/m^2^; LVRI, SD=0.34g/ml; and LVSI, SD=0.087). *Indicates p<0.05. Abbreviations are as in Table-2.


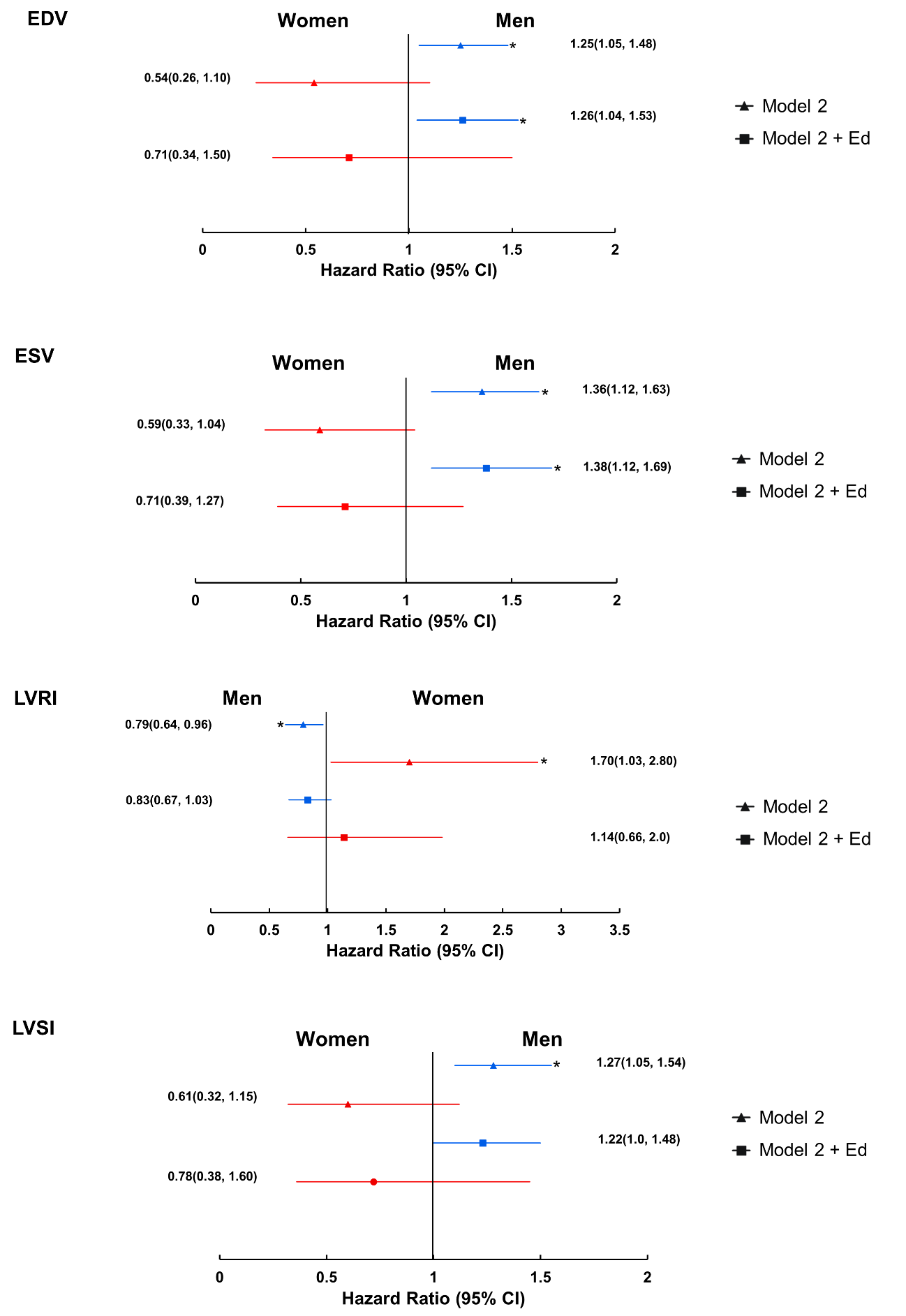


**A**


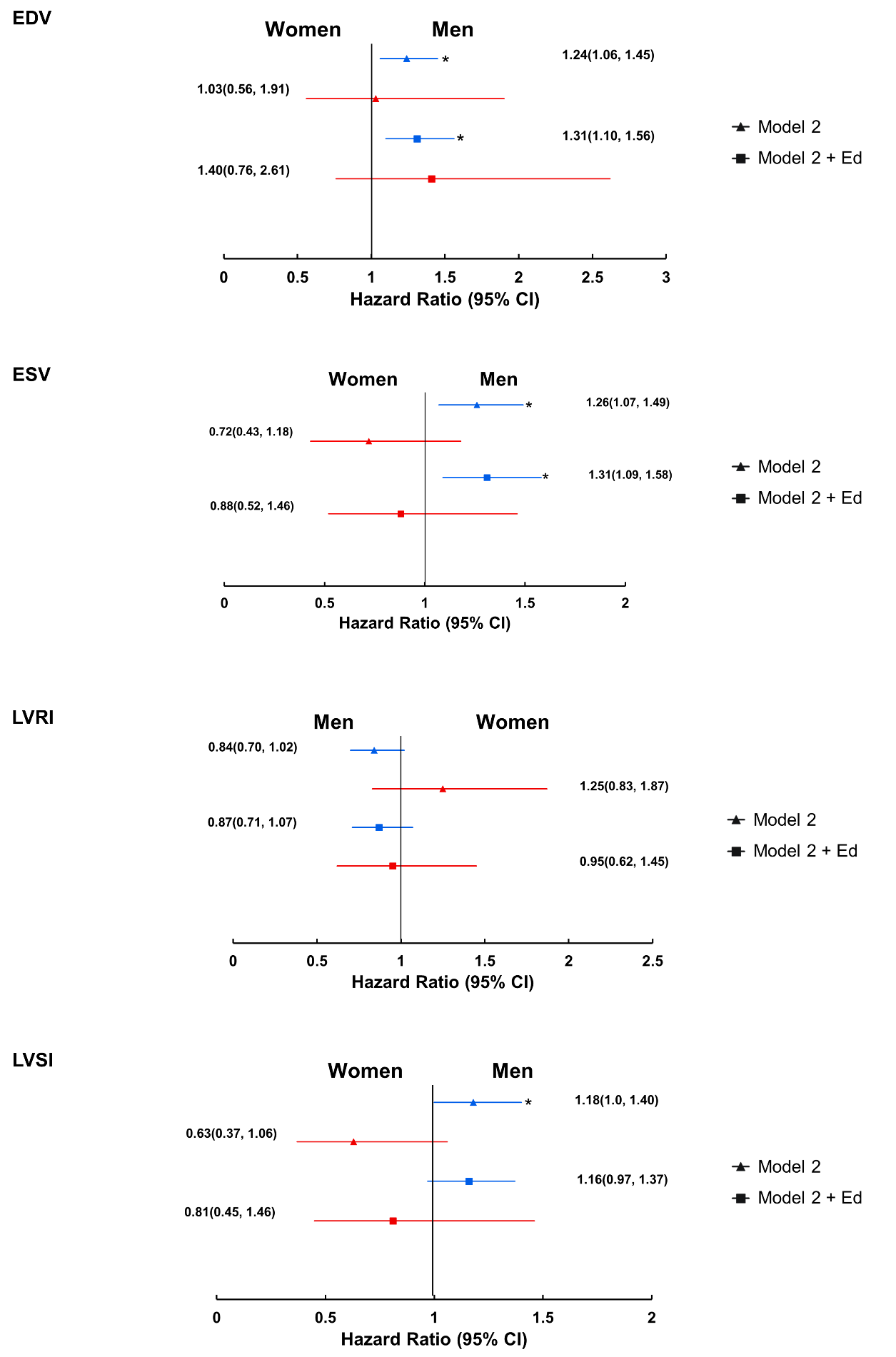


**B**

**Figure-2 Forest plots showing the associations between 3DE LV measures and all-cause mortality(A) and composite cardiovascular endpoint(B) stratified by sex; role of diastolic function(imputed data).** *Indicates p<0.05. Abbreviations: Ed, LV diastolic stiffness. Remaining abbreviations as in Table-2. Footnotes as in Figure-1.


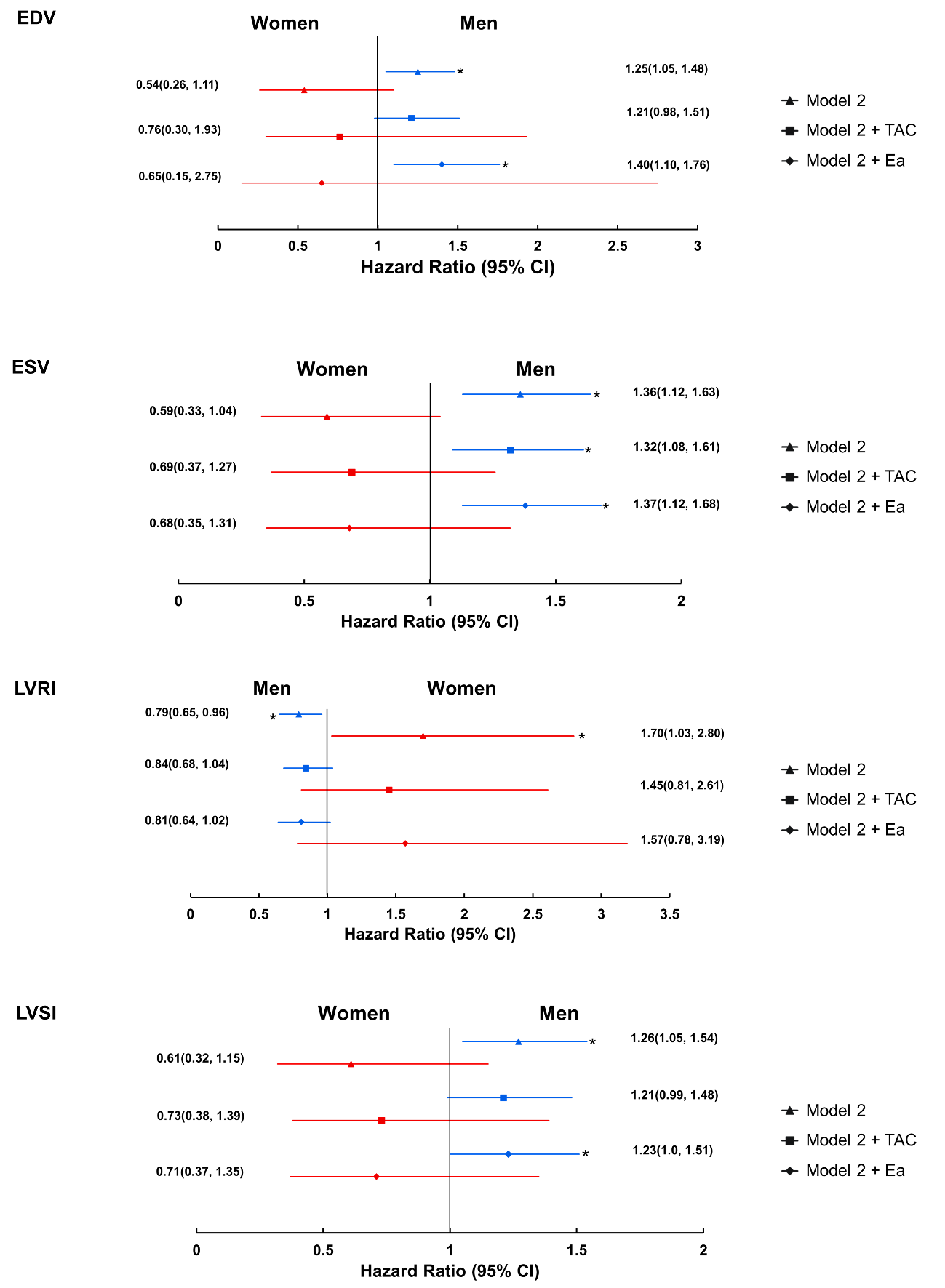


**A**


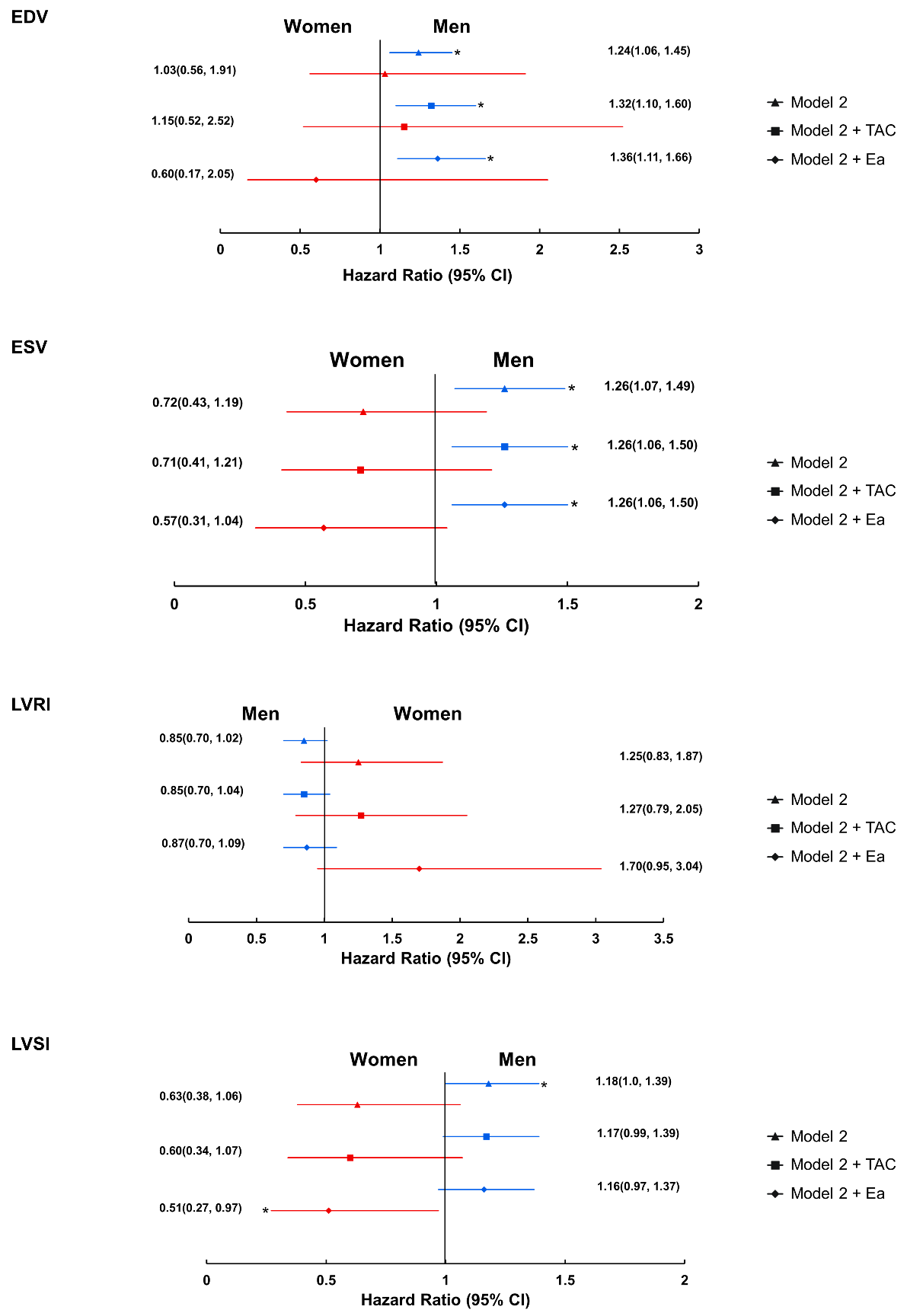


**B**

**Figure-3 Forest plots showing the associations between 3DE-derived LV measures and all-cause mortality(A) and composite cardiovascular endpoint(B) stratified by sex; role of macro-vascular disease(imputed data).** *Indicates p<0.05. Abbreviations: Ea, effective arterial elastance; and TAC, total arterial compliance. Remaining abbreviations as in Table-2. Footnotes as in Figure-1.

**
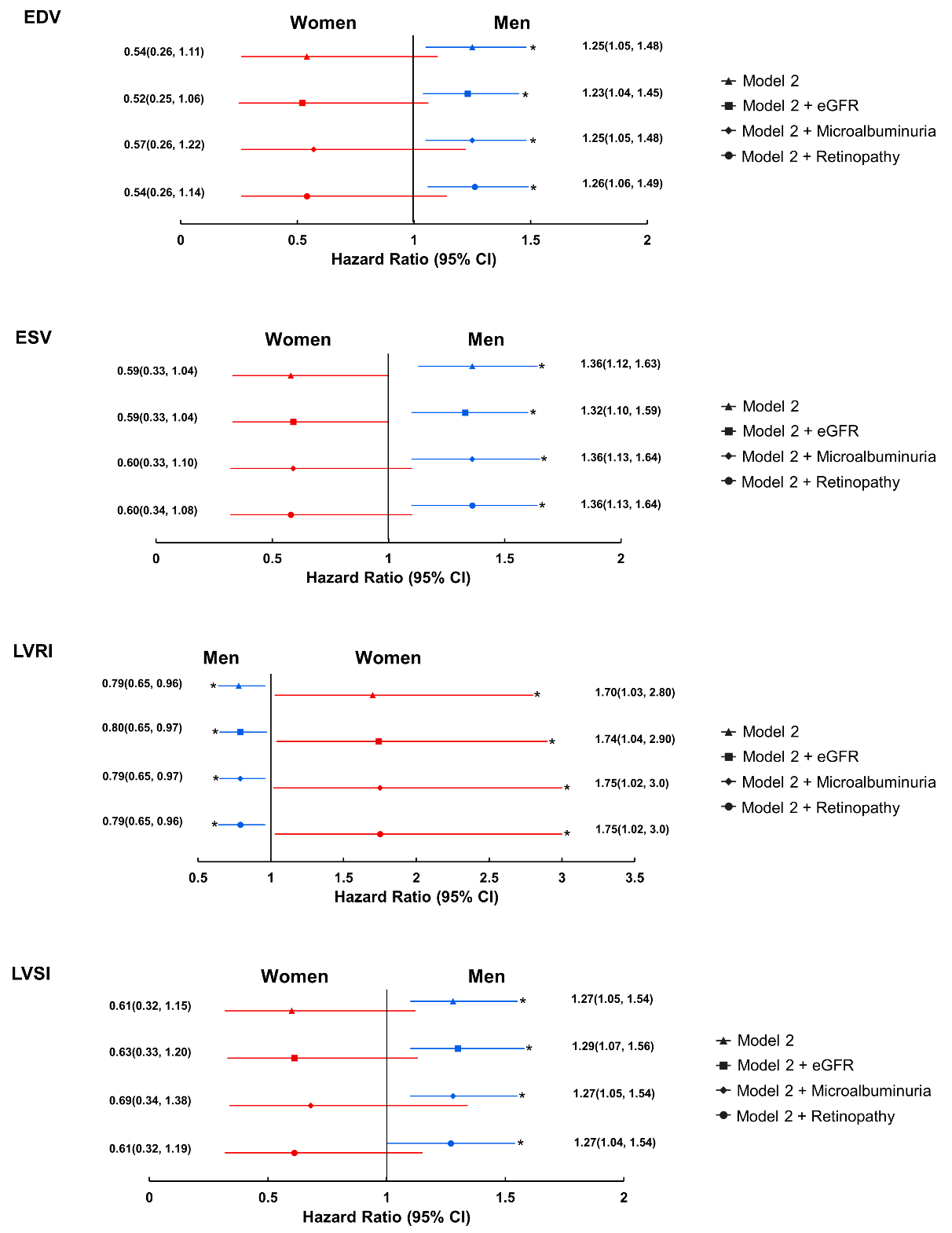
**

**A**


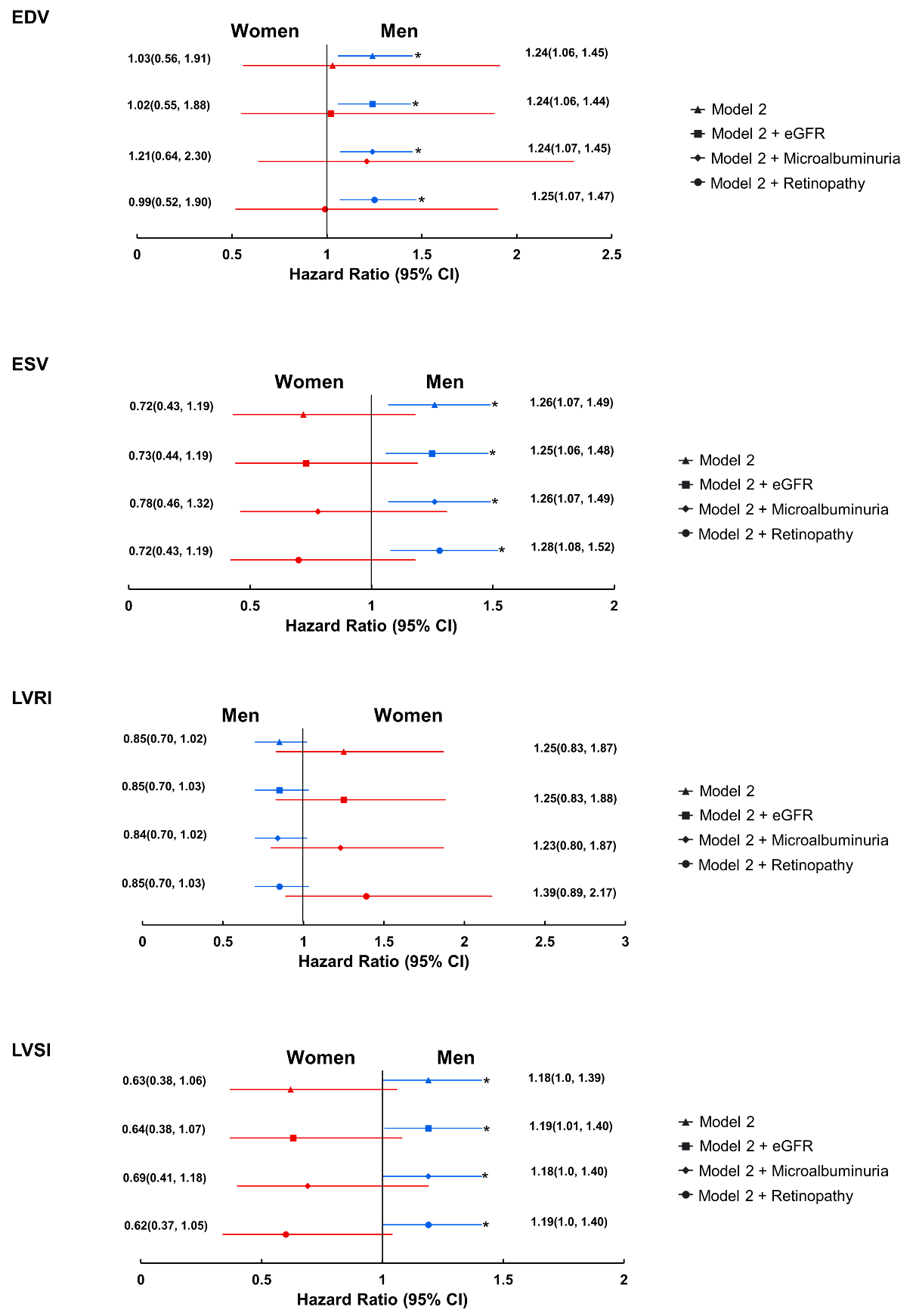


**B**

**Figure-4 Forest plots showing the associations between 3DE-derived LV measures and all-cause mortality(A) and composite cardiovascular endpoint(B) stratified by sex; role of micro-vascular disease(imputed data).** *Indicates p<0.05. Abbreviations: eGFR, estimated glomerular filtration rate. Remaining abbreviations as in Table-2. Footnotes as in Figure-1.
