# Supplementary material for: Sex-differences in associations of LV structure and function measured by echocardiography with long-term risk of mortality and cardiovascular morbidity
Source: Front Cardiovasc Med. 2023 Apr 25;10:1144964. doi: 10.3389/fcvm.2023.1144964 (PMC10166834; doi:10.3389/fcvm.2023.1144964)
Supplement: Supplementary file 3 [file Table3.docx]

15 January 2023

**LONDON’S GLOBAL UNIVERSITY**

**INSTITUTE OF CARDIOVASCULAR SCIENCE**

## Sebastian Kelle

## Specliaty Chief Editor, Frontiers in Cardiovascular Medicine

Dear Professor Kelle

**Re: Sex-differences in LV structure and function by 3D echocardiography in associations with long-term risk of mortality and cardiovascular morbidity**

We would be grateful for your consideration of the above manuscript for publication in the *Frontiers in Cardiovascular Medicine Journal.*

3D echocardiography(3DE) imaging is currently recommended by the ASE as a method of choice for assessing LV structure and function. Despite this, there is limited evidence regarding its usefulness in the general population. We show that altered cardiac function and structure(and shape) assessed by 3DE are associated with the long-term risk of all-cause mortality and cardiovascular morbidity in a large sample of community dwelling older people(SABRE study). However, the direction of several of these associations differs by sex. Increased LV volumes and LV sphericity index and decreased LV remodeling index(LVRI) and EF were associated with increased risk of all-cause mortality in men, but associations were null or reversed in women. Although numbers of women were small, effect sizes indicated that lower LV volumes and higher LVRI were associated with higher risk of all-cause mortality in women. We also show that these sex-differences seemed to be partially explained by higher ventricular stiffness(i.e. worse diastolic function) and arterial stiffness in women. This is consistent with evidence from HF patients, where LV systolic dysfunction and eccentric remodeling/hypertrophy carry prognostic information in men, while diastolic function and LV concentric remodeling/hypertrophy may be more important for risk in women. Hence, these results might have important clinical implications for early prevention of disease and initiation of therapeutic interventions to improve prognosis before disease progression in individuals at particular risk from the general population.

All authors contributed to this study. LA, ADH and NC conceived and designed the study. ADH, JM, DF and NC handled funding. LA, SJ and CP performed the data collection. LA performed the statistical analysis assisted by TT and ADH. CP, ADH and TT assisted in data interpretation. LA drafted the manuscript and all co-authors critically reviewed, amended and approved the manuscript.

We confirm that the paper is not under consideration elsewhere, none of the paper's contents have previously been published except as abstracts presented in scientific conferences, all authors have read and approved the manuscript, and we have fully disclosed our potential conflicts of interests.

We wish to propose the following as possible reviewers who have unique and extensive experience on the subject:

**Phil Chowiencyzk**

**Rosa Maria Bruno**

**Petros** **Nihoyannopoulos**

Tom Marwick is conflicted as he was an PhD examiner for LA.

Yours sincerely,

Alun Hughes, corresponding author on behalf of all authors.
